# Prognostic Assessment of Oxidative Stress-Related Genes in Colorectal Cancer and New Insights into Tumor Immunity

**DOI:** 10.1155/2022/2518340

**Published:** 2022-10-15

**Authors:** Zilu Chen, Kun Mei, Yao Xiao, Yan Xiong, Wei Long, Qin Wang, Jiang Zhong, Dongmei Di, Yunxi Ge, Yi Luo, Ziyun Li, Yan Huang, Renjun Gu, Bin Wang

**Affiliations:** ^1^Nanjing University of Chinese Medicine, Nanjing 210023, China; ^2^Department of Cardiothoracic Surgery, The Third Affiliated Hospital of Soochow University, Changzhou 213003, China; ^3^Department of Ultrasound, Nanjing Hospital of Chinese Medicine Affiliated to Nanjing University of Chinese Medicine, Nanjing 210001, China; ^4^Department of Oncology, Affiliated Hospital of Integrated Traditional Chinese and Western Medicine, Nanjing University of Chinese Medicine, Nanjing, Jiangsu 210028, China; ^5^Department of Oncology, Jiangsu Province Hospital on Integration of Chinese and Western Medicine, Nanjing, Jiangsu 210028, China; ^6^School of Acupuncture and Tuina, School of Regimen and Rehabilitation, Nanjing University of Chinese Medicine, Nanjing 210023, China; ^7^School of Chinese Medicine & School of Integrated Chinese and Western Medicine, Nanjing University of Chinese Medicine, Nanjing 210023, China

## Abstract

Oxidative stress is crucial to the biology of tumors. Oxidative stress' potential predictive significance in colorectal cancer (CRC) has not been studied; nevertheless here, we developed a forecasting model based on oxidative stress to forecast the result of CRC survival and enhance clinical judgment. The training set was chosen from the transcriptomes of 177 CRC patients in GSE17536. For validation, 65 samples of colon cancer from GSE29621 were utilized. For the purpose of choosing prognostic genes, the expression of oxidative stress-related genes (OXEGs) was found. Prognostic risk models were built using multivariate Cox regression analysis, univariate Cox regression analysis, and LASSO regression analysis. The outcomes of the western blot and transcriptome sequencing tests were finally confirmed. ATF4, CARS2, CRP, GPX1, IL1B, MAPK8, MRPL44, MTFMT, NOS1, OSGIN2, SOD2, AARS2, and FOXO3 were among the 14 OXEGs used to build prognostic characteristics. Patients with CRC were categorized into low-risk and high-risk groups according on their median risk scores. Cox regression analysis using single and multiple variables revealed that OXEG-related signals were independent risk factors for CRC. Additionally, the validation outcomes from western blotting and transcriptome sequencing demonstrated that OXEGs were differently expressed. Using 14 OXEGs, our work creates a predictive signature that may be applied to the creation of new prognostic models and the identification of possible medication candidates for the treatment of CRC.

## 1. Introduction

Colorectal cancer (CRC) is a common malignant tumor of the gastrointestinal tract [1]. It is the second most lethal malignancy in China, after lung cancer, with an incidence of roughly 40,800 persons. The fourth malignant tumor accounts for around 195,600 annual deaths [2]. Currently, colorectal cancer patients can receive treatment through surgery, endoscopic procedures, radiotherapy, chemotherapy, and immunotherapy [3], but the overall survival rate of colorectal cancer patients has not dramatically enhanced [4]. On account of the high invasiveness and paucity of awareness of early physical examination, diagnosis is often made once symptoms have advanced or the disease has metastasized, which poses significant challenges for the prognosis and course of treatment.

Tumor development consists of a multitude of complex variables. The oxidative stress factor plays a significant role in numerous stages of tumor advancement, including the transformation of normal cells into tumor cells, proliferation, tumor angiogenesis, and metastasis [5–8]. The term “tumor of oxidative stress” refers to an improper regulation mechanism of oxidative signaling and oxidative damage of macromolecules caused by an imbalance in the body's oxidation and antioxidation system of mutual limitation [9].

The effect of oxidative stress mainly involves reactive nitrogen species (RNS) and reactive oxygen species (ROS) as an outcome of chemical reactions; the resulting effect is often seen as a double-edged sword, with great controversy over the tumor-promoting and tumor-suppressing effects [6, 10], the specific effect of ROS levels on tumor cells themselves, [11] sensitivity and lack of oxygen, and the tumor microenvironment of regulatory factors. When the content of ROS is insufficient to break the balance between oxidative and antioxidant systems in the tumor growth environment, it can participate in the regulation of epithelial-mesenchymal transition (EMT), tumor angiogenesis, and other processes by activating PI3K/Akt and NF-*κ*B signaling pathways [12–14]. ROS can also promote the metastasis and proliferation of tumor cells [15]. At the same time, low levels of ROS can cause occasional DNA base mismatch and DNA damage in the body, which will be repaired immediately. However, when a large amount of ROS causes the amassing of DNA damage in tumor cells to the extent that it cannot be repaired, conventional base excision repair [16] and nucleotide excision repair cannot remove the damaged DNA. Thus, the tumor cells have to undergo programmed cell death.

Plasma medicine is an emerging academic area that combines clinical medicine, physics, and life sciences. Cold atmospheric plasma-activated medium (PAM), which is dependent on plasma production of active substances transferred to the medium, has a wide range of uses [17]. Its anticancer effect is generally considered to be through ROS and RNS [18–20]. Therefore, PAM was selected as an oxidative stress source to verify the relationship between oxidative stress and tumor prognosis.

In recent years, the relationship between the accumulation of ROS and immunotherapy has been more and more frequently mentioned. It is particularly important to clarify the technique of the body's immune feedback to a tumor and the escape of tumor cells from immune effect during oxidative stress [21, 22]. ROS production also affects the anticancer effects of CD8+ and CD4+ T cells in the tumor microenvironment [23, 24]. Many clinical trials and successful checkpoint immunotherapy instances have shown the crucial role that the cellular immune system plays in the treatment of cancer.

In the process of oxidative stress and colorectal cancer research, early prediction models and prognostic gene screening are particularly important. In this research, based on information from Gene Expression Omnibus (GEO), related genes that may affect the prognosis of CRC were studied.

## 2. Materials and Methods

### 2.1. Data Sources and Processing

The CRC cohort's transcriptional dataset with aligning clinical data were downloaded from the Gene Expression Omnibus (GEO) database. After comprehensive screening, data sets GSE17536 and GSE29621 were chosen for this study, in which 177 patients with symptoms of colorectal cancer were included in GSE17536 as a training set, and 65 patients with colorectal cancer were included in GSE29621 as the validation set.

### 2.2. Screening for Oxidative Stress-Related Genes

Eighty genes related to oxidative stress were retrieved from GeneCards (https://www.genecards.org/). The cut-offs were set as relevance score > 20 (Supplement 1). Subsequently, 77 expressed genes of oxidative stress were identified by Venn diagram package (1.7.1).

### 2.3. Establishment and Assessment of Prognostic Risk Score Model

Univariate Cox regression screening was performed for prognostic OXEGs. The LASSO algorithm was implemented to find the value of the minimum error of cross validation and obtain the best prognostic gene of the model. Finally, stable OXEGs were constructed as the final prognostic model. Kaplan-Meier curves were utilized to establish prognostic differences between groups, and ROC curves were used to calculate the 1-, 3-, and 5-year survival of patients. Finally, the correlation between patients' low-risk and high-risk groups and clinical information was calculated.

### 2.4. Nomogram Prognosis Prediction Model Establishment

We used the “RMS” package in version R (4.2.0) to plot the lipopograph model in combination with patient's age, sex, grade, stage, and risk score. To show the accord between the actual survival probabilities at 1, 3, and 5 years and those predicted by the nomogram, calibration curves were developed. Finally, the model was validated using the ROC curve, multivariate Cox regression, and univariate Cox regression.

### 2.5. Correlation between Low-Risk and High-Risk Groups for Immune Cell Infiltration

CIBERSORT R package was used to establish the amount of tumor-infiltrating immune cells in CRC tumor samples. Finally, we examined the functional differences of tumor immune cells through “reshape2,” “GSVA,” and “GSEABase” software package in R version (4.2.0).

### 2.6. Gene Enrichment Examination between High-Risk and Low-Risk Groups

In order to show the influence of potential biological pathways in the differential expression of OXEGs, r-packet clusterProfiler was utilized for gene ontology (GO) enrichment examination and KEGG pathway examination. The molecular signatures database (MSigDB) used with the Gene Set Enrichment Analysis (GSEA) software version (holdings) was C2 (C2. Cp. Kegg. 7.5.1. Entrez. GMT) used to evaluate the link between biological and genetic traits. The significance level was set to *P* < 0.05, and the number of arbitrary sample permutations was set to 1000.

### 2.7. Transcriptome Sequencing Validation of Colorectal Cancer

Three CRC tissues from the Anorectal Department, Nanjing Hospital of Traditional Chinese Medicine were collected and matched with normal tissues for transcriptome sequencing. Patients received no neoadjuvant chemotherapy or radiotherapy. Consent was obtained from the study participants prior to study commencement. Clinicopathological characteristics of these patients were also collected. The collected tissues were frozen in liquid nitrogen. The Declaration of Helsinki, the World Medical Association's code of ethics for human experimentation, was followed throughout the performance of this study. The ethics committee of the Nanjing Hospital of Traditional Chinese Medicine had to approve the study. Informed consent was signed by all patients whose tissue samples were collected before the study began.

### 2.8. Cell Line Culture and Treatment

The CRC cell line SW480 was grown in 1640 media with 10% fetal bovine serum (FBS), 1% penicillin/streptomycin, and 5% carbon dioxide in a humid environment at 37°C. All materials for cell culture were purchased from Gibco, USA. Cell lines within 10 generations were selected to reduce the influence of passage on experimental results. The particular plasma jet was designed by Nanjing Tech University. PAM is made by plasma jet spraying PBS solution at a distance of 5 mm. In the control group, 200 *μ*l of 50% PAM solution was added to the medium and grown for 24 hours.

### 2.9. Enzyme-Linked Immunosorbent Assay (ELISA)

Each group's culture medium supernatant was collected. Each batch of cells' supernatant was examined using ELISA kits from Quanzhou Ruixin Biological Technology Co., Ltd. in Quanzhou, China, to measure the amount of IL-17A components present.

### 2.10. Cell Transfection

The SW480 ACT1-knockdown cells were transfected using LipofectamineTM 2000 Transfection Reagent (11668019). The Supplement 2 document includes the siRNA sequence screening. Six-well plates containing SW80 cells were planted with 5,105 cells per well. 250 l of Opti-MEM was added to two EP tubes once the cells had acquired a confluency of 60–70%. Then, 5 *μ*g siRNA was added to one tube and 5 *μ*l Lipofectamine 2000 to the other tube. After mixing, the tubes were left at noncold normal temperature for 5 min and then, the liquid of the two EP tubes was mixed gently and placed on an ultraclean table for 20 min, followed by the incubator for 6 h before changing to complete medium and continuing to culture. The fresh medium was replaced after 24 hours, and 200 *μ*l of 50% PAM solution was added to the medium and cultured for 24 hours.

### 2.11. Western Blotting

RIPA lysis buffer (Epizyme Biomedical Technology, Shanghai, China) was used to extract total proteins in SW480 on ice (with 1% protease and phosphatase inhibitors). Before the samples were differentiated using 12% SDS-PAGE and conveyed to the polyvinylidene difluoride (PVDF) membranes, the total protein content was evaluated using the BCA Protein Assay Kit (TransGen Biotech, Beijing, China). After 15 minutes of blocking with QuickBlock Blocking Buffer from Beyotime Biotechnology in Shanghai, China, membranes were grown with various diluted primary antibodies overnight at 4°C. Following three TBST washes, the membranes were incubated for 1 hour with a second antibody that had been diluted. Proteintech (Wuhan, China) provided the ACT1 (26692-1-AP) primary antibody, while Abmart Technology provided the TRAF6 (T55175S), NF-B (p65) (T55034S), and MAPK P38 (T40075S) primary antibodies (Shanghai, China). The secondary antibody, goat antirabbit IgG (H + L) HRP (BL003A), was bought from Biosharp (Hefei, China).

### 2.12. Statistical Analysis

GraphPad Prism 8.0 was used to conduct all statistical analyses (GraphPad Software, San Diego, CA, USA). The Student *t*-test was used to compare the variations between the means of the two groups. The mean and standard deviation for all statistical data were displayed (SD). Statistics were identified as significant when *P* < 0.05.

## 3. Results

### 3.1. Prognostic Risk Signature Construction of OXEGs

**Figure 1:**
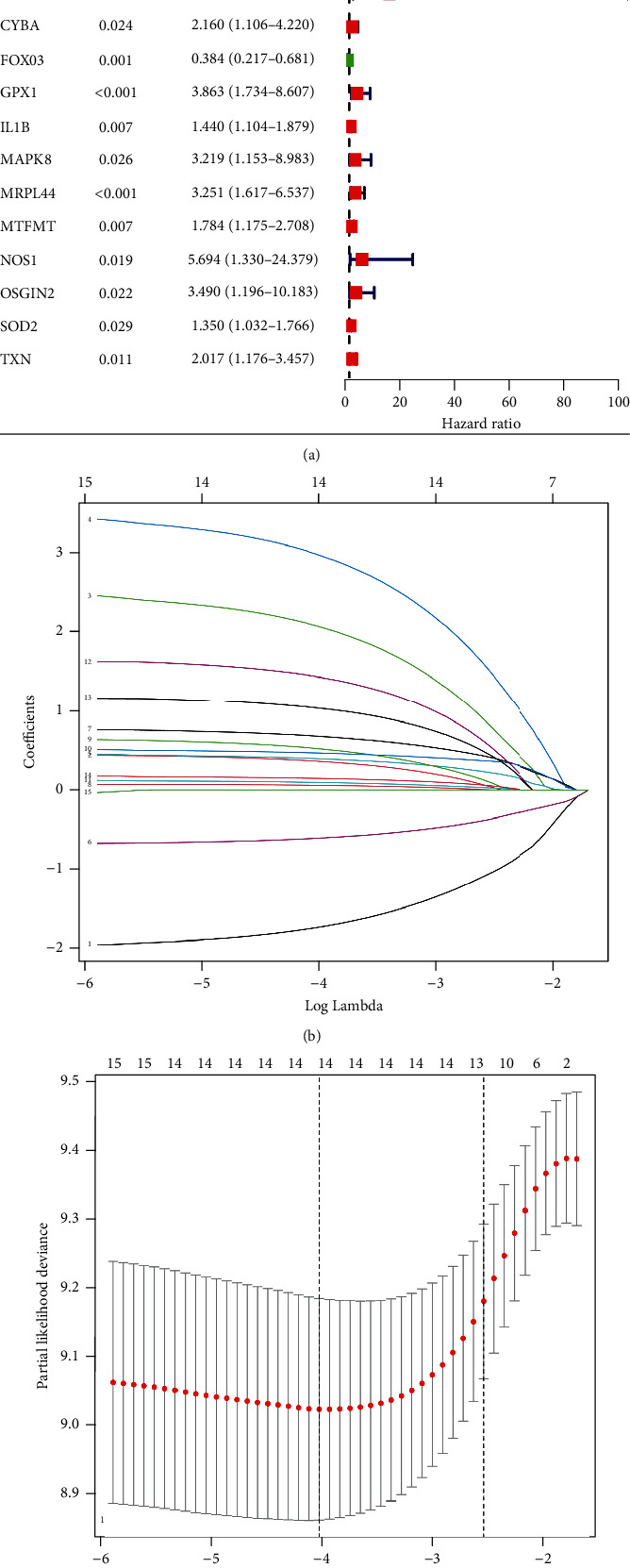
Construction of the prognostic model in GEO-CRC. (a) According to the results of univariate Cox regression analysis, a total of 15 genes were identified as prognostic genes; (b) LASSO coefficient profiles of the prognostic genes; and (c) turning optimal parameter (lambda) screening in the LASSO model.

**Figure 2:**
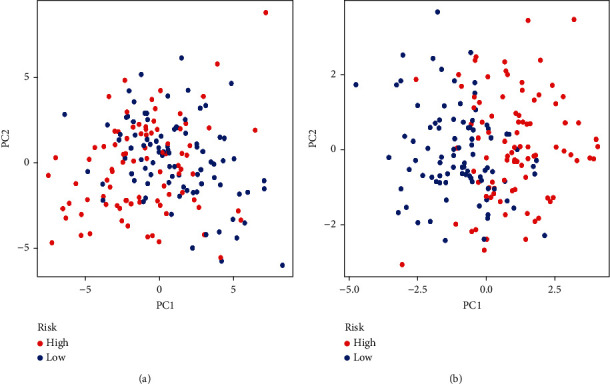
Principal component analysis. (a) PCA analysis was performed in high- and low-risk groups. (b) PCA analysis of prognosis model.

Based on univariate Cox regression analysis, 15 genes were recognized as prognostic (Figure 1(a)). Finally, we screened 14 features of OSDEGs analysis after LASSO analysis and multivariate Cox regression analysis (Figures 1(b) and 1(c)). AARS2 and FOXO3 were the protective factors in the prognostic model, while ATF4, CARS2, CRP, CYBA, GPX1, IL1B, MAPK8, MRPL44, MTFMT, NOS1, OSGIN2, and SOD2 were considered as risk factors in the prognostic model. The risk score for each CRC patient in GSE17536 was assessed using the following equation: Risk score = (−1.74 × AARS2 expression) + (0.36 × ATF4 expression) + (2.08 × CARS2 expression) + (2.98 × CRP expression) + (0.40 × CYBA expression) + (−0.61 × FOXO3 expression) + (0.68 × GPX1 expression) + (0.06 × IL1B expression) + (0.52 × MAPK8 expression) + (0.47 × MRPL44 expression) + (0.09 × MTFMT expression) + (1.43 × NOS1 expression) + (1.04 × OSGIN2 expression) + (0.14 × SOD2 expression). Finally, CRC patients in GSE17536 were categorized as the high-risk and the low-risk groups based on the median risk score. We performed PCA analysis on both groups (Figures 2(a) and 2(b)) and found that the prognostic model genes could effectively identify between the low-risk and high-risk categories.

### 3.2. Evaluation of the Prognostic Performance of the OXEGs Signature

**Figure 3:**
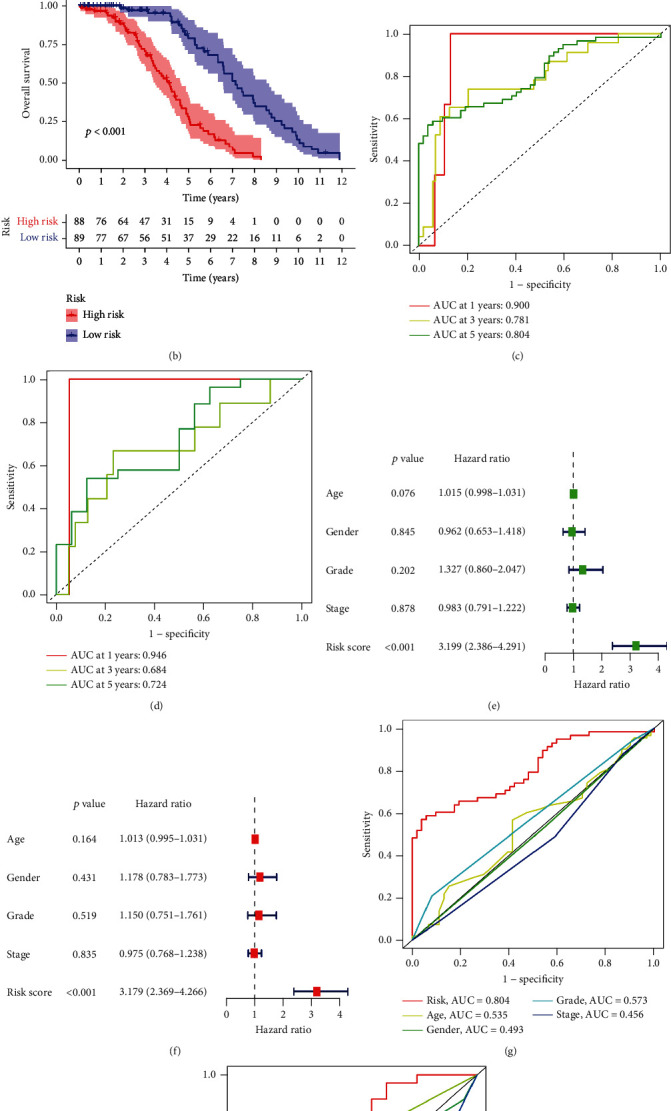
Evaluation of the prognostic signature in GEO-CRC. (a) Kaplan-Meier (K-M) curve of overall survival for the training set; (b) K-M curves of overall survival for the validation set; (c) ROC curves of 1-year, 3-year, and 5-year survival rates for the training set; (d) ROC curves of 1-year, 3-year, and 5-year survival rates for the validation set; (e, f) Cox regression analysis of risk scores and other clinical characteristics (age, gender, grade, stage); (g) ROC curve of clinical characteristics for the training set; and (h) ROC curve of clinical characteristics for the validation set.

**Figure 4:**
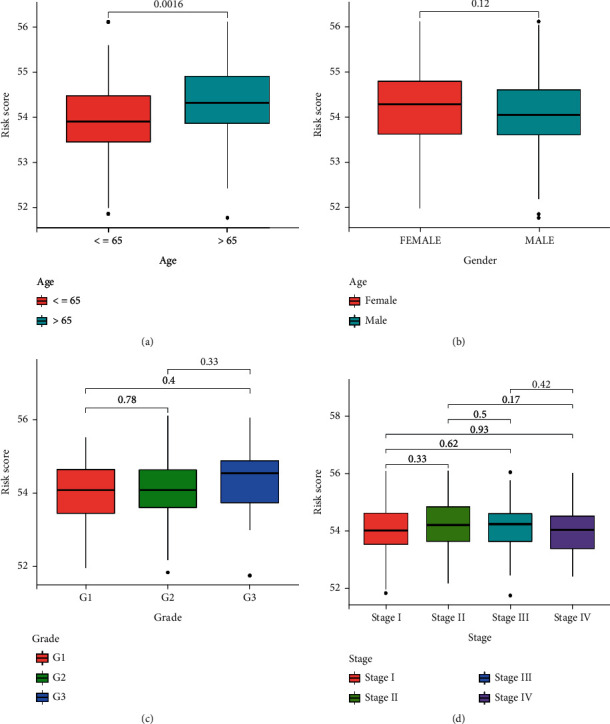
Relationship between the risk groups and clinical features in GEO-CRC. (a) Correlation between risk score and age; (b) correlation between risk score and gender; (c) correlation between risk score and grade; and (d) correlation between risk score and stage.

The low-risk group had a finer prognosis and a longer surviving time, whereas the high-risk group had an inferior prognosis and a shorter survival time, as shown by the Kaplan-Meier survival curve (Figure 3(a)). Additionally, we used the external validation dataset GSE29621 to confirm the prognostic risk profile's accuracy, and we found consistent variation in overall survival (OS) between the high-risk and the low-risk groups. (Figure 3(b)). Area under the curve (AUC) measurements for the 1-, 3-, and 5-year survival rates were 0.900, 0.781, and 0.804, respectively, while these measurements for the 1-, 3-, and 5-year AUC of the validation set were 0.946, 0.684, and 0.724, respectively. This data demonstrated that our prognostic prediction had good sensitivity and specificity (Figures 3(c) and 3(d)). Subsequently, we showed from Cox regression analysis that the prognostic risk models associated with OXEGs are independent predictors of CRC prognosis (Figures 3(e) and 3(f)). We further predicted the relationship between clinical features and prognostic value by ROC (Figure 3(g)), and the AUC of the model was 0.804, while the AUC of age, gender, stage, and grade were 0.535, 0.493, 0.573, and 0.456, respectively, demonstrating the model's excellent sensitivity and specificity. The ROC curve of the validation set signified that the AUC value of the model was 0.724, which was higher than other clinical features (Figure 3(h)). Through clinical correlation analysis (Figures 4(a)–4(d)), we concluded that age was substantially different between the groups at high- and low-risk (*P* < 0.05), while gender, grade, and stage were not statistically significant.

### 3.3. Nomogram Construction and Evaluation

**Figure 5:**
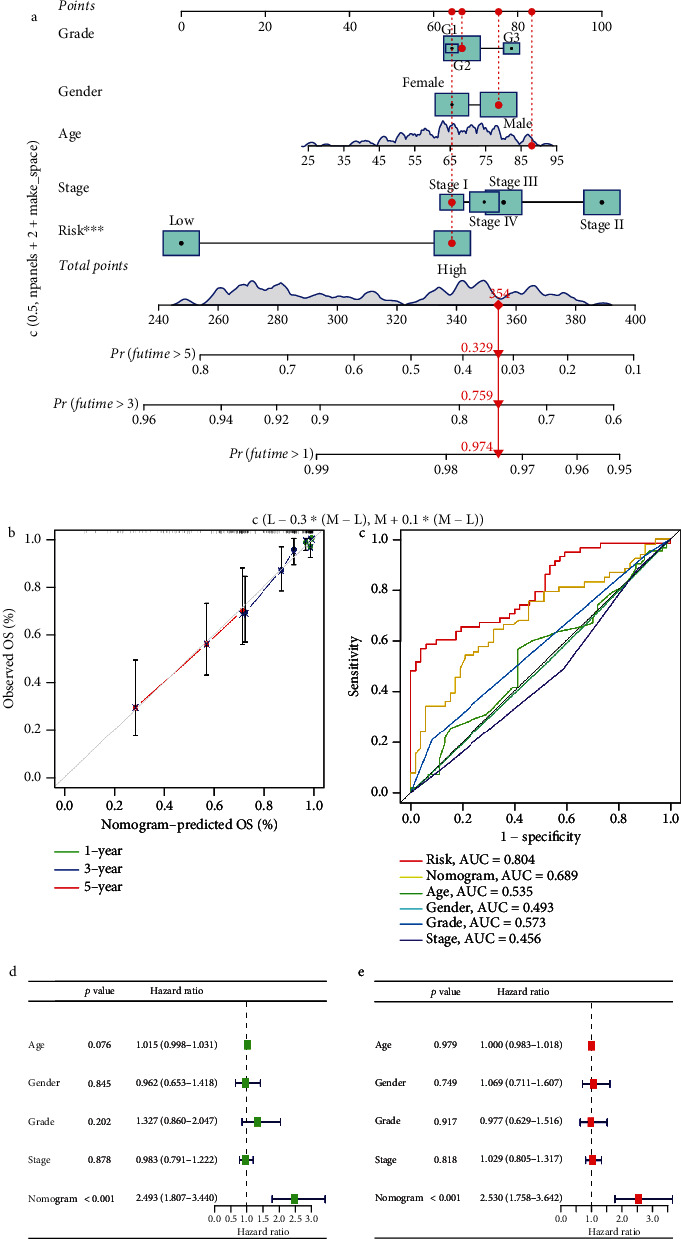
Nomogram prediction model and evaluation. (a) Nomogram of age, sex, stage, stage, and risk score for predicting 1-, 3-, and 5-year survival. (b) 1-, 3-, and 5-year calibration curves for the GEO dataset; (c) ROC curve of nomogram; (d) Cox regression analysis of risk score and other clinical characteristics of nomogram (age, gender, grade, stage).

We constructed a nomogram based on age, sex, grade, stage, and risk scores for 14 OXEGs to foretell the 1-, 3-, and 5-year survival in CRC patients (Figure 5(a)). As can be seen from the calibration curve (Figure 5(b)), the survival prediction for year 5 is in good agreement with the actual value. In the ROC curve (Figure 5(c)), the value of AUC was 0.689, indicating that the model had high accuracy. The rosette model was later demonstrated to be an independent determinant of CRC prognosis by univariate and multivariate Cox regression analysis. (Figures 5(d) and 5(e)).

### 3.4. Association between Tumor Immune Cell Infiltration and Risk Score

**Figure 6:**
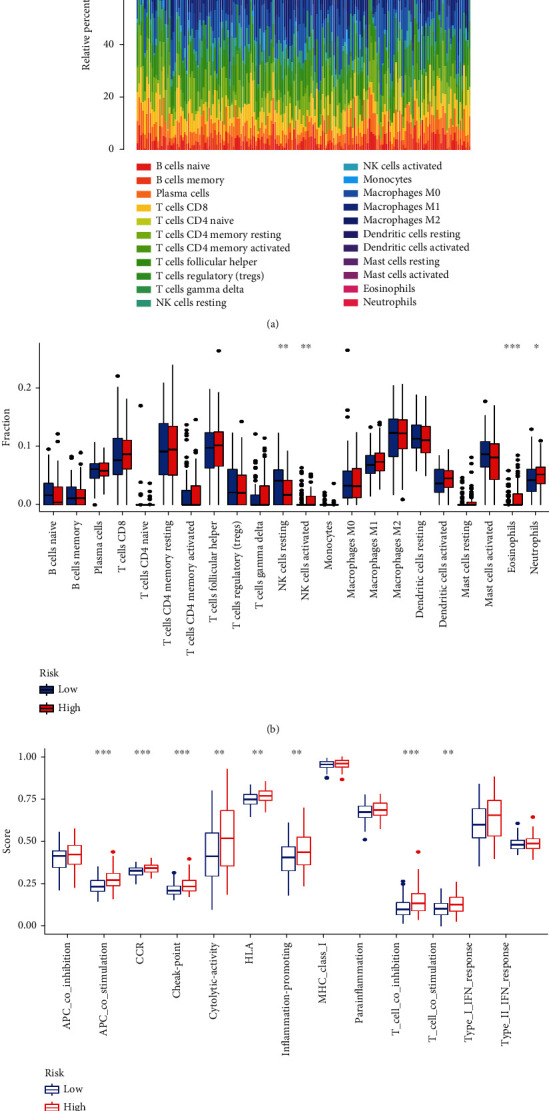
Immunoinfiltration analysis. (a) Tumor infiltrating immune cell distribution map; (b) correlation between risk score model and tumor infiltrating immune cells; (c) correlation between risk score model and tumor infiltrating immune cell function. ^∗^*P* < 0.05; ^∗∗^*P* < 0.01; ^∗∗∗^*P* < 0.001.

Calculations were made to determine the variation in immune cells between the low-risk group and the high-risk group using the CIBERSORT method to get the waterfall diagram of immune cells in the tumor group (Figures 6(a) and 6(b)). As seen in the image, the high-risk group had elevated levels of neutrophils, eosinophils, and activated NK cells (*P* < 0.05), but the low-risk group had considerably higher levels of resting NK cells infiltrating their tissues. APC costimulation, inflammation-promoting, CCR, T cell costimulation, cytolytic activity, HLA, T cell coinhibition, and checkpoint were all more significant in the high-risk group, according to the analyses of immune cell function (Figure 6(c)).

### 3.5. Gene Enrichment Analysis

**Figure 7:**
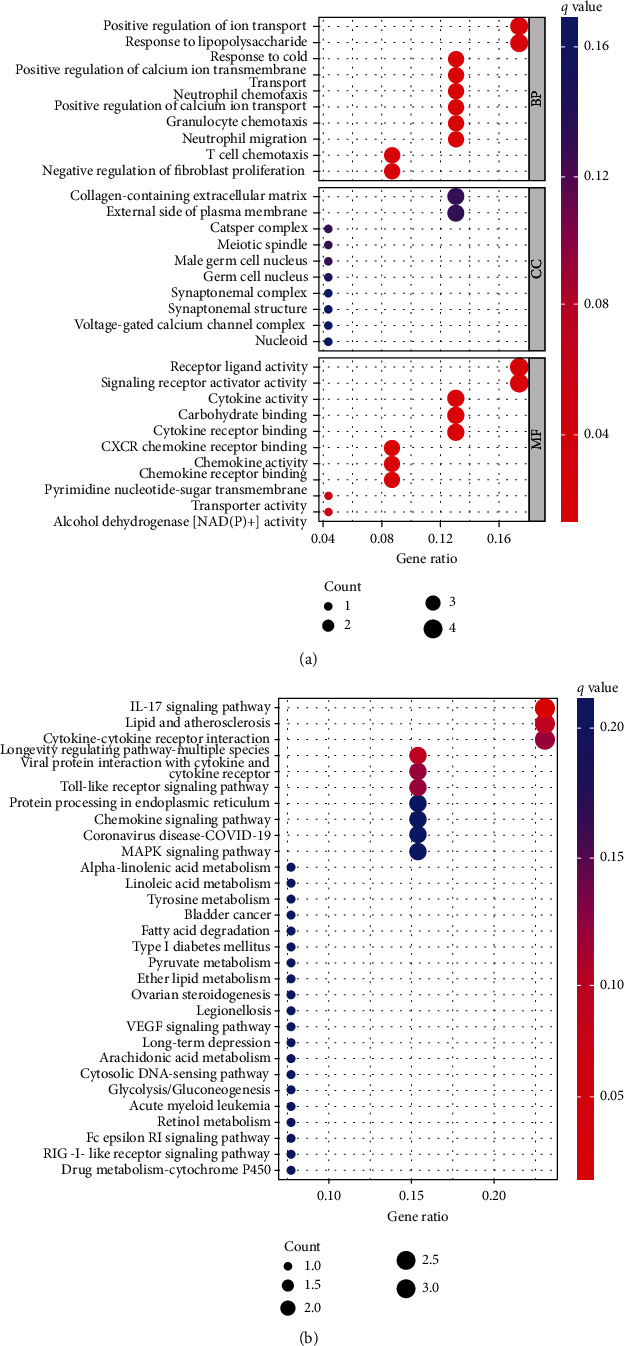
GO and KEGG enrichment analyses. (a) GO enrichment analysis; (b) KEGG enrichment analysis.

**Figure 8:**
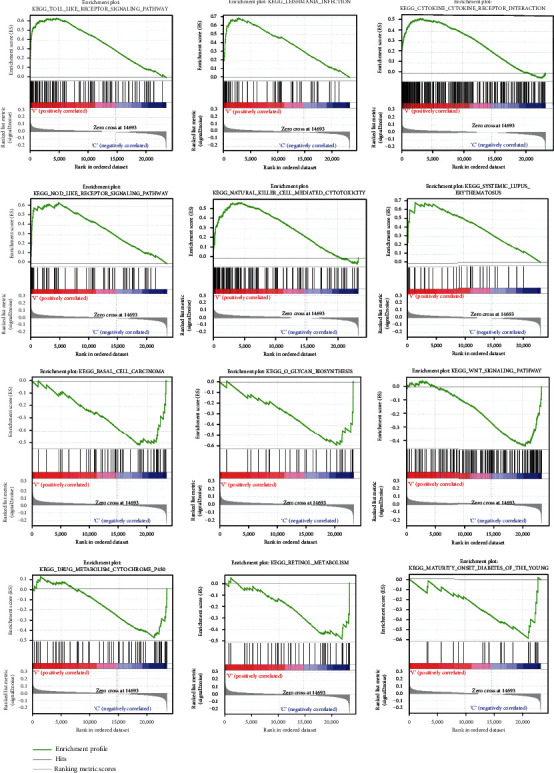
GSEA enrichment analysis.

Twenty-seven differential genes were identified in the low-risk and high-risk categories (Supplement 3). We examined the GO and KEGG pathways of the differential genes and obtained a total of 68 KEGG signaling pathways and 832 significantly enriched biological processes (Supplements 4 and 5). The biological processes were enriched for positive control of ion transport, response to lipopolysaccharide, response to cold, positive control of calcium ion transmembrane transport, neutrophil chemotaxis, positive management of calcium ion transport, granulocyte migration, and T cell chemotaxis. In the cellular component, collagen-regulation of fibroblast extracellular matrix, external side of plasma membrane, CatSper complex, meiotic spindle, and male germ cell nucleus were enriched. In addition, we demonstrated significant enrichment of receptor ligand activity, signaling receptor activator activity, cytokine activity, carbohydrate binding, cytokine receptor binding, CXCR chemokine receptor binding, and chemokine activity in molecular function (Figure 7(a)). The IL-17 signaling pathway, lipid and atherosclerosis, cytokine-cytokine receptor interaction, longevity regulating pathway-multiple species, viral protein interaction with cytokine and cytokine receptor, and Toll-like receptor signaling pathway were the pathways that KEGG enrichment analysis showed to be enriched (Figure 7(b)). The enrichment disparities between the high-risk and the low-risk groups may be seen by GSEA. Systemic lupus erythematosus, basal cell carcinoma, O-glycan biosynthesis, Wnt signaling pathway, retinol metabolism, drug metabolism cytochrome p450, and maturity-onset diabetes of the young were the major areas of enrichment in the low-risk group. Cytokine receptor interaction, leishmania infection, NOD-like receptor signaling pathway, natural killer cell mediated cytotoxicity, and systemic lupus were the major areas of enrichment in the high-risk group (Figure 8).

### 3.6. Validation of Prognostic Genes and Enrichment Pathways

**Figure 9:**
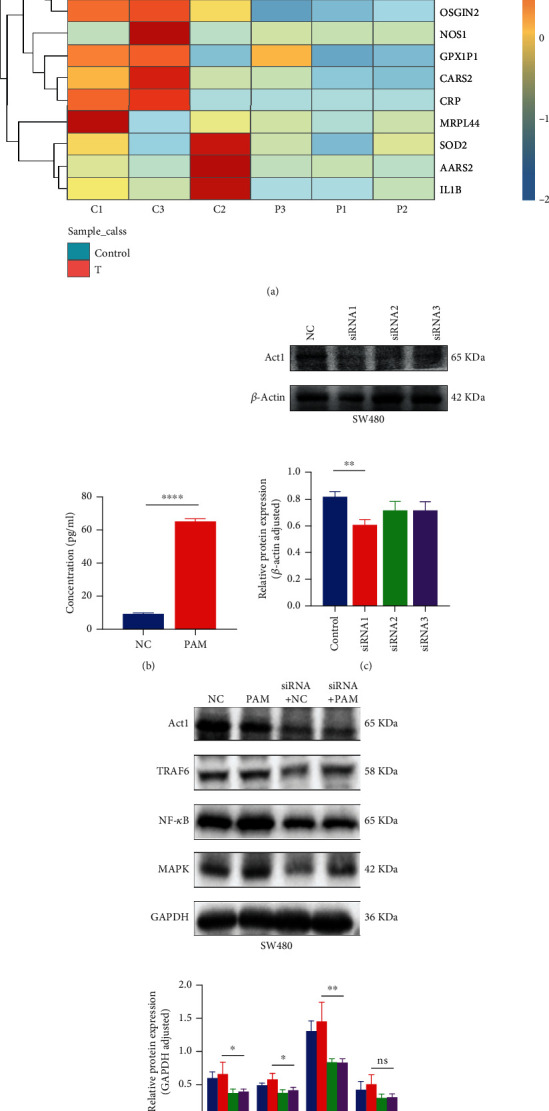
Validation of prognostic genes and enrichment pathways. (a) Prognostic gene sequencing heat map; (b) the increased expression of IL-17 in SW480 cell line was verified by ELISA; (c) validation of ACT1 knockdown in SW480 cell line; (d) validation of IL-17 pathway proteins. ^∗^*P* < 0.05; ^∗∗^*P* < 0.01; ^∗∗∗^*P* < 0.001.

Sequencing results portrayed that prognostic genes were different in colorectal cancer tissues and adjacent tissues. As can be seen from Figure 9, FOXO3 and MAPK8 were highly expressed in adjacent tissues. ATF4, MTFMT, CYBA, OSGIN2, NOS1, GPX1P1, CARS2, CRP, MRPL44, SOD2, AARS2, and IL-1B are highly expressed in colorectal cancer tissues (Figure 9(a)). After ELISA confirmed that IL-17A was increased in the oxidative stimulation group (Figure 9(b)), we knocked down the IL-17A receptor ACT1 (Figure 9(c)) and performed oxidative stimulation again. Western blot results signify the expressions of downstream signaling factors TRAF6, NF-*κ*B, and MAPK signaling pathways were decreased to a certain extent after ACT1 knockdown (Figure 9(d)).

## 4. Discussion

As one of the prevalent malignant tumors in the world, colorectal cancer has a high fatality rate. The 5-year survival rate for individuals with advanced CRC is around 14%, and more than 50% of patients receive their diagnosis at an advanced stage [25, 26]. There is still an urgency for developing a prognostic model in order to give tailored prediction and precision medicine for CRC patients who are dealing with the therapeutic conundrum.

In the body, the content of ROS in normal cells and tumor cells is different, and the sensitivity to oxidative stress is also different. Oxidative DNA damage has been widely accepted as an important feature of the occurrence of malignant tumors. At present, researchers have found a variety of novel treatment methods based on ROS to restore chemoresistance and overcome radiotherapy resistance, enhance the efficacy of chemoradiotherapy, [27–31] and bring certain guiding significance to clinical treatment. At the same time, oxidative stress products in the tumor microenvironment also affect the immune response of the body. Therefore, we established and verified the prognostic design in relation to OXEGs to foretell the prognosis of CRC patients.

Using multivariate and univariate Cox regression analysis with the LASSO technique, we examined the differentially expressed genes related to oxidative stress in GEO. 14 OXEGs were screened (AARS2, FOXO3, ATF4, CARS2, CRP, CYBA, GPX1, IL1B, MAPK8, MRPL44, MTFMT, NOS1, OSGIN2, and SOD2) to create a prognostic risk model for prediction. The model we created has good specificity and sensitivity and ROC testing showed that its AUC was 0.804. Additionally, we discovered that age was a predictive factor that affected CRC patients on its own. AARS2 and FOXO3 are protective factors in the prognostic model of CRC, and the former regulates the proliferation of colorectal cancer by affecting mitochondrial respiration [32]. The latter is associated with morbidity and mortality in CRC [33] and can modulate its mediated SOX2 expression to affect cancer migration, invasion, and stem cell proliferation [34]. ATF4 regulates tumor autophagy in CRC and affects tumor survival [35]. CRP is related to systemic inflammation, but its effect on colorectal cancer is not clear. CYBA can induce familial colorectal cancer by interfering with the integrity of intestinal barrier [36]. IL-1B, as a member of the interleukin family, is closely related to tumor immunity [37]. SOD2 contributes to the chemical resistance of colorectal cancer [38], and according to research, MAPK8 can promote the progression of colorectal cancer [39]. However, the functional roles of CARS2, GPX1, MRPL44, MTFMT, NOS1, and OSGIN2 in CRC are still unknown. The sensitivity and accuracy of this model are further verified by the combined GEO dataset. Our findings suggest that higher risk is associated with poorer outcomes.

A total of 68 KEGG signaling pathways and 832 significantly enriched biological processes were obtained through enrichment analysis. The most interesting point was the significant enrichment of the IL-17 pathway. When ROS content increased, levels of the proinflammatory cytokine IL-17A increased through retardation of the PI3K/AKT/mTOR pathway and selective autophagy [40]. We also demonstrated by ELISA that IL-17A levels were significantly increased in SW480 cells after oxidative stimulation. Many studies have also proven the relationship between IL-17A level and REDOX environment [41, 42]. To this end, we performed western blot verification of oxidative stimulation after knocking down the receptor ACT1 of IL-17A [43] and found that the downstream factor TRAF6 and the relative content of corresponding pathways including NF-*κ*B and MAPK were affected (see mechanism diagram in the Supplementary Material). MAPK may not be statistically significant due to the interference of other related pathways, which will be discussed further.

PAM can increase the content of ROS in tumor cells, and the accumulation of a large number of active substances leads to tumor cell death. Many researchers have affirmed the effect of PAM in the treatment of tumors in vivo and in vitro. Nakamura et al. [44] showed that PAM inhibited the metastasis of ovarian cancer through in vivo and in vitro experiments, and Utsumi et al. proved that PAM also has a certain therapeutic effect on chemotherapy resistant ovarian cancer [45]. To expand on the selection of PAM concentration in this study, we will further select a wider range of concentrations to verify the difference between low concentration and high concentration groups.

We estimated the variation in immune infiltration between the two categories using the CIBERSORT method in order to study the link between immune cell infiltration and risk ratings. We identified significant increases in monocyte, activated NK cells, eosinophils, and neutrophil levels in the low-risk group. Moreover, the largely enriched IL-17A pathway is closely linked to immune cells. Bruno et al. attenuated the antifungal host immune response by using IL-17A inhibitors, which increased the incidence of Candida infection [46]. These results suggest that oxidative stress-related gene tags may influence immune cell infiltration and hence the efficacy of colorectal cancer immunotherapy.

## 5. Conclusions

In conclusion, using 14 OXEGs, we created a prognostic model for colorectal cancer under oxidative stress that has a high predictive value. This study provides the possibility for individuals with CRC to get individualized care.

## Data Availability

The data used to support the findings of this study are available from the corresponding author upon request.

## Supplementary Materials

Supplementary 1 Supplement 1: 80 genes related to oxidative stress.

Supplementary 2 Supplement 2: Three ACT1 knockdown siRNA sequences were used for screening.

Supplementary 3 Supplement 3: Table 3: Twenty-seven differential genes were identified in the low-risk and high-risk groups.

Supplementary 4 Supplement 4: Table 4: GO enrichment analysis.

Supplementary 5 Supplement 5: Table 4: KEGG enrichment analysis.
