# Supplementary material for: Prognostic Assessment of Oxidative Stress-Related Genes in Colorectal Cancer and New Insights into Tumor Immunity
Source: Oxid Med Cell Longev. 2022 Oct 15;2022:2518340. doi: 10.1155/2022/2518340 (PMC9590115; doi:10.1155/2022/2518340)
Supplement: Supplementary 1 — Supplement 1: 80 genes related to oxidative stress. [file 2518340.f1.docx]

80 genes related to oxidative stress

| NOS3 | NOS2 | NOS1 | SOD1 | CAT | CPT2 | AIFM1 | CARS2 | TNF | ELAC2 | FARS2 | NFE2L2 | TP53 | HMOX1 | OXSR1 | GFM1 |
| --- | --- | --- | --- | --- | --- | --- | --- | --- | --- | --- | --- | --- | --- | --- | --- |
| AARS2 | SOD2 | MAPK14 | GSR | OSGIN1 | MAPK8 | XDH | OSER1 | MPO | IL6 | TXN | TUFM | OSGIN2 | POLR1C | MAPK1 | TSFM |
| PARK7 | OLR1 | MTRFR | IL1B | G6PD | TXN2 | VARS2 | PNPT1 | SIRT1 | CYCS | CASP3 | RYR2 | GPX1 | NQO1 | APP | CRP |
| MTFMT | PTGS2 | PON1 | CXCL8 | HSPA5 | GTPBP3 | ACADVL | MAP3K5 | ADPRS | SLC6A4 | NOS1AP | CRH | HADHA | MRPL44 | CYBA | CALM1 |
| FOXO3 | JUN | GSTM1 | CCL2 | INS | HADHB | G3BP1 | OGG1 | XBP1 | DDIT3 | GFM2 | FOXO1 | TARS2 | ATF4 | RYR1 | PRDX2 |
