# Supplementary material for: Prognostic Assessment of Oxidative Stress-Related Genes in Colorectal Cancer and New Insights into Tumor Immunity
Source: Oxid Med Cell Longev. 2022 Oct 15;2022:2518340. doi: 10.1155/2022/2518340 (PMC9590115; doi:10.1155/2022/2518340)
Supplement: Supplementary 2 — Supplement 2: Three ACT1 knockdown siRNA sequences were used for screening. [file 2518340.f2.doc]

Three ACT1 knockdown siRNA sequences were used for screening

| gene | species | Sequence | | |
| --- | --- | --- | --- | --- |
|  |  | Target sequance | sense（5'-3'） | antisense（5'-3'） |
| H10758-siAct1-1 | Human | CACCCTGTGCAGAAGGTTA | CACCCUGUGCAGAAGGUUA(dT)(dT) | UAACCUUCUGCACAGGGUG(dT)(dT) |
| H10758-siAct1-2 | Human | CTCCATGGAACTATCATTA | CUCCAUGGAACUAUCAUUA(dT)(dT) | UAAUGAUAGUUCCAUGGAG(dT)(dT) |
| H10758-siAct1-3 | Human | GGATGAGCATGGCTTACAT | GGAUGAGCAUGGCUUACAU(dT)(dT) | AUGUAAGCCAUGCUCAUCC(dT)(dT) |
