# Supplementary material for: Prognostic Assessment of Oxidative Stress-Related Genes in Colorectal Cancer and New Insights into Tumor Immunity
Source: Oxid Med Cell Longev. 2022 Oct 15;2022:2518340. doi: 10.1155/2022/2518340 (PMC9590115; doi:10.1155/2022/2518340)
Supplement: Supplementary 3 — Supplement 3: Table 3: Twenty-seven differential genes were identified in the low-risk and high-risk groups. [file 2518340.f3.docx]

Twenty-seven differential genes were identified in the low-risk and high-risk groups

| gene | lowMean | highMean | logFC | pValue | fdr |
| --- | --- | --- | --- | --- | --- |
| ADH1C | 7.835753 | 7.30825 | -0.10055 | 0.005663 | 0.032618 |
| AK025288 | 8.243225 | 7.671898 | -0.10363 | 1.82E-05 | 0.000856 |
| BC017398 | 6.907685 | 6.26375 | -0.14118 | 0.001745 | 0.015432 |
| BCL2A1 | 7.524393 | 8.281011 | 0.138232 | 1.29E-05 | 0.0007 |
| CD69 | 6.965112 | 7.537489 | 0.113937 | 0.000181 | 0.003661 |
| CHP2 | 8.220303 | 7.639159 | -0.10578 | 1.68E-05 | 0.000819 |
| CLC | 6.849775 | 7.544773 | 0.139421 | 7.31E-06 | 0.000511 |
| CLEC4A | 7.007843 | 7.52642 | 0.102993 | 7.36E-08 | 3.44E-05 |
| CPE | 8.635876 | 8.038034 | -0.1035 | 0.000435 | 0.006324 |
| CXCL10 | 9.543404 | 10.27061 | 0.105946 | 0.000901 | 0.01008 |
| CXCL11 | 7.365393 | 8.275057 | 0.168007 | 0.003103 | 0.022308 |
| DACH1 | 8.221348 | 7.631977 | -0.10732 | 0.001122 | 0.011587 |
| HCAR3 | 6.811427 | 7.468693 | 0.132899 | 8.50E-05 | 0.002289 |
| HLA-DQA1 | 7.785056 | 8.374648 | 0.105321 | 0.000405 | 0.006037 |
| HMCN1 | 7.418494 | 6.913148 | -0.10178 | 0.006651 | 0.036094 |
| HSPA2 | 8.227933 | 7.605795 | -0.11343 | 0.001918 | 0.016399 |
| HSPA4L | 6.228124 | 7.047523 | 0.178319 | 4.99E-06 | 0.00043 |
| LRRN1 | 4.991483 | 4.635716 | -0.10668 | 0.008569 | 0.042338 |
| MMP1 | 9.513483 | 10.27906 | 0.111662 | 0.004019 | 0.026198 |
| PLA2G4A | 7.133494 | 7.705432 | 0.111267 | 0.005012 | 0.03017 |
| RARRES3 | 8.633011 | 9.309216 | 0.108796 | 8.29E-05 | 0.002249 |
| RETNLB | 8.473292 | 7.701477 | -0.13779 | 0.002086 | 0.017264 |
| S100A8 | 6.737719 | 7.268466 | 0.109391 | 3.86E-07 | 9.14E-05 |
| SLC35D3 | 7.362191 | 6.70958 | -0.13391 | 0.005389 | 0.031609 |
| SOD2 | 7.115876 | 7.718807 | 0.117336 | 6.67E-08 | 3.27E-05 |
| TNFSF9 | 6.768899 | 7.354261 | 0.119659 | 3.93E-06 | 0.000374 |
| ZIC2 | 6.185371 | 7.112057 | 0.201407 | 0.000462 | 0.006591 |
