# Supplementary material for: Prognostic Assessment of Oxidative Stress-Related Genes in Colorectal Cancer and New Insights into Tumor Immunity
Source: Oxid Med Cell Longev. 2022 Oct 15;2022:2518340. doi: 10.1155/2022/2518340 (PMC9590115; doi:10.1155/2022/2518340)
Supplement: Supplementary 4 — Supplement 4: Table 4: GO enrichment analysis. [file 2518340.f4.docx]

GO enrichment analysis

| ONTOLOGY | ID | Description | pvalue | p.adjust | qvalue | geneID |
| --- | --- | --- | --- | --- | --- | --- |
| BP | GO:0009409 | response to cold | 3.21E-05 | 0.022247 | 0.015286 | CXCL10/HSPA2/SOD2 |
| BP | GO:1904427 | positive regulation of calcium ion transmembrane transport | 8.66E-05 | 0.030046 | 0.020644 | CXCL10/CXCL11/HSPA2 |
| BP | GO:0030593 | neutrophil chemotaxis | 0.000284 | 0.043554 | 0.029925 | CXCL10/CXCL11/S100A8 |
| BP | GO:0043270 | positive regulation of ion transport | 0.00031 | 0.043554 | 0.029925 | CXCL10/CXCL11/HSPA2/PLA2G4A |
| BP | GO:0051928 | positive regulation of calcium ion transport | 0.000399 | 0.043554 | 0.029925 | CXCL10/CXCL11/HSPA2 |
| BP | GO:0071621 | granulocyte chemotaxis | 0.000494 | 0.043554 | 0.029925 | CXCL10/CXCL11/S100A8 |
| BP | GO:1990266 | neutrophil migration | 0.000494 | 0.043554 | 0.029925 | CXCL10/CXCL11/S100A8 |
| BP | GO:0010818 | T cell chemotaxis | 0.000531 | 0.043554 | 0.029925 | CXCL10/CXCL11 |
| BP | GO:0048147 | negative regulation of fibroblast proliferation | 0.00061 | 0.043554 | 0.029925 | DACH1/SOD2 |
| BP | GO:0032496 | response to lipopolysaccharide | 0.000656 | 0.043554 | 0.029925 | CXCL10/CXCL11/S100A8/SOD2 |
| BP | GO:1904064 | positive regulation of cation transmembrane transport | 0.000754 | 0.043554 | 0.029925 | CXCL10/CXCL11/HSPA2 |
| BP | GO:0002237 | response to molecule of bacterial origin | 0.000824 | 0.043554 | 0.029925 | CXCL10/CXCL11/S100A8/SOD2 |
| BP | GO:0097530 | granulocyte migration | 0.000846 | 0.043554 | 0.029925 | CXCL10/CXCL11/S100A8 |
| BP | GO:1903169 | regulation of calcium ion transmembrane transport | 0.000879 | 0.043554 | 0.029925 | CXCL10/CXCL11/HSPA2 |
| BP | GO:0034767 | positive regulation of ion transmembrane transport | 0.000998 | 0.046152 | 0.031711 | CXCL10/CXCL11/HSPA2 |
| BP | GO:0051281 | positive regulation of release of sequestered calcium ion into cytosol | 0.001086 | 0.047088 | 0.032354 | CXCL10/CXCL11 |
| BP | GO:0009266 | response to temperature stimulus | 0.001328 | 0.052928 | 0.036366 | CXCL10/HSPA2/SOD2 |
| BP | GO:0046006 | regulation of activated T cell proliferation | 0.001373 | 0.052928 | 0.036366 | CLC/TNFSF9 |
| BP | GO:0050798 | activated T cell proliferation | 0.001561 | 0.057004 | 0.039167 | CLC/TNFSF9 |
| BP | GO:0010043 | response to zinc ion | 0.001899 | 0.062122 | 0.042683 | S100A8/SOD2 |
| BP | GO:0050832 | defense response to fungus | 0.001899 | 0.062122 | 0.042683 | CLEC4A/S100A8 |
| BP | GO:0010524 | positive regulation of calcium ion transport into cytosol | 0.001971 | 0.062122 | 0.042683 | CXCL10/CXCL11 |
| BP | GO:0010332 | response to gamma radiation | 0.002118 | 0.062122 | 0.042683 | CXCL10/SOD2 |
| BP | GO:0034764 | positive regulation of transmembrane transport | 0.002148 | 0.062122 | 0.042683 | CXCL10/CXCL11/HSPA2 |
| BP | GO:0051651 | maintenance of location in cell | 0.002325 | 0.064536 | 0.044342 | CXCL10/CXCL11/S100A8 |
| BP | GO:0097529 | myeloid leukocyte migration | 0.002639 | 0.068316 | 0.046939 | CXCL10/CXCL11/S100A8 |
| BP | GO:0009620 | response to fungus | 0.002756 | 0.068316 | 0.046939 | CLEC4A/S100A8 |
| BP | GO:0048247 | lymphocyte chemotaxis | 0.002756 | 0.068316 | 0.046939 | CXCL10/CXCL11 |
| BP | GO:0030595 | leukocyte chemotaxis | 0.002873 | 0.068755 | 0.047241 | CXCL10/CXCL11/S100A8 |
| BP | GO:0072678 | T cell migration | 0.003016 | 0.069773 | 0.047941 | CXCL10/CXCL11 |
| BP | GO:0051924 | regulation of calcium ion transport | 0.003418 | 0.076519 | 0.052576 | CXCL10/CXCL11/HSPA2 |
| BP | GO:0048145 | regulation of fibroblast proliferation | 0.004166 | 0.086733 | 0.059594 | DACH1/SOD2 |
| BP | GO:0061844 | antimicrobial humoral immune response mediated by antimicrobial peptide | 0.004166 | 0.086733 | 0.059594 | CXCL10/CXCL11 |
| BP | GO:0051279 | regulation of release of sequestered calcium ion into cytosol | 0.004269 | 0.086733 | 0.059594 | CXCL10/CXCL11 |
| BP | GO:0048144 | fibroblast proliferation | 0.004374 | 0.086733 | 0.059594 | DACH1/SOD2 |
| BP | GO:0033273 | response to vitamin | 0.004587 | 0.088435 | 0.060763 | CXCL10/SOD2 |
| BP | GO:0070098 | chemokine-mediated signaling pathway | 0.005256 | 0.098033 | 0.067358 | CXCL10/CXCL11 |
| BP | GO:0097193 | intrinsic apoptotic signaling pathway | 0.005368 | 0.098033 | 0.067358 | BCL2A1/S100A8/SOD2 |
| BP | GO:1990868 | response to chemokine | 0.006212 | 0.106352 | 0.073074 | CXCL10/CXCL11 |
| BP | GO:1990869 | cellular response to chemokine | 0.006212 | 0.106352 | 0.073074 | CXCL10/CXCL11 |
| BP | GO:0070588 | calcium ion transmembrane transport | 0.00638 | 0.106352 | 0.073074 | CXCL10/CXCL11/HSPA2 |
| BP | GO:0060326 | cell chemotaxis | 0.006436 | 0.106352 | 0.073074 | CXCL10/CXCL11/S100A8 |
| BP | GO:0008630 | intrinsic apoptotic signaling pathway in response to DNA damage | 0.006717 | 0.108416 | 0.074492 | BCL2A1/SOD2 |
| BP | GO:0010522 | regulation of calcium ion transport into cytosol | 0.006977 | 0.110048 | 0.075613 | CXCL10/CXCL11 |
| BP | GO:0051235 | maintenance of location | 0.007377 | 0.113766 | 0.078168 | CXCL10/CXCL11/S100A8 |
| BP | GO:0009408 | response to heat | 0.007783 | 0.116087 | 0.079762 | CXCL10/HSPA2 |
| BP | GO:0010038 | response to metal ion | 0.008662 | 0.116087 | 0.079762 | CHP2/S100A8/SOD2 |
| BP | GO:1904062 | regulation of cation transmembrane transport | 0.00873 | 0.116087 | 0.079762 | CXCL10/CXCL11/HSPA2 |
| BP | GO:0045471 | response to ethanol | 0.009216 | 0.116087 | 0.079762 | S100A8/SOD2 |
| BP | GO:0072676 | lymphocyte migration | 0.009216 | 0.116087 | 0.079762 | CXCL10/CXCL11 |
| BP | GO:0051209 | release of sequestered calcium ion into cytosol | 0.009515 | 0.116087 | 0.079762 | CXCL10/CXCL11 |
| BP | GO:0019730 | antimicrobial humoral response | 0.009667 | 0.116087 | 0.079762 | CXCL10/CXCL11 |
| BP | GO:0051283 | negative regulation of sequestering of calcium ion | 0.009667 | 0.116087 | 0.079762 | CXCL10/CXCL11 |
| BP | GO:0051282 | regulation of sequestering of calcium ion | 0.009973 | 0.116087 | 0.079762 | CXCL10/CXCL11 |
| BP | GO:0051208 | sequestering of calcium ion | 0.010598 | 0.116087 | 0.079762 | CXCL10/CXCL11 |
| BP | GO:0050900 | leukocyte migration | 0.011057 | 0.116087 | 0.079762 | CXCL10/CXCL11/S100A8 |
| BP | GO:0010944 | negative regulation of transcription by competitive promoter binding | 0.01217 | 0.116087 | 0.079762 | DACH1 |
| BP | GO:0033483 | gas homeostasis | 0.01217 | 0.116087 | 0.079762 | SOD2 |
| BP | GO:0060245 | detection of cell density | 0.01217 | 0.116087 | 0.079762 | DACH1 |
| BP | GO:0006986 | response to unfolded protein | 0.012404 | 0.116087 | 0.079762 | HSPA2/HSPA4L |
| BP | GO:0055076 | transition metal ion homeostasis | 0.012404 | 0.116087 | 0.079762 | S100A8/SOD2 |
| BP | GO:0010959 | regulation of metal ion transport | 0.012594 | 0.116087 | 0.079762 | CXCL10/CXCL11/HSPA2 |
| BP | GO:0010212 | response to ionizing radiation | 0.012918 | 0.116087 | 0.079762 | CXCL10/SOD2 |
| BP | GO:0008611 | ether lipid biosynthetic process | 0.013379 | 0.116087 | 0.079762 | PLA2G4A |
| BP | GO:0042118 | endothelial cell activation | 0.013379 | 0.116087 | 0.079762 | CXCL10 |
| BP | GO:0046504 | glycerol ether biosynthetic process | 0.013379 | 0.116087 | 0.079762 | PLA2G4A |
| BP | GO:0060081 | membrane hyperpolarization | 0.013379 | 0.116087 | 0.079762 | SOD2 |
| BP | GO:0071492 | cellular response to UV-A | 0.013379 | 0.116087 | 0.079762 | MMP1 |
| BP | GO:0090084 | negative regulation of inclusion body assembly | 0.013379 | 0.116087 | 0.079762 | HSPA2 |
| BP | GO:0090481 | pyrimidine nucleotide-sugar transmembrane transport | 0.013379 | 0.116087 | 0.079762 | SLC35D3 |
| BP | GO:0090527 | actin filament reorganization | 0.013379 | 0.116087 | 0.079762 | HMCN1 |
| BP | GO:0097384 | cellular lipid biosynthetic process | 0.013379 | 0.116087 | 0.079762 | PLA2G4A |
| BP | GO:0007584 | response to nutrient | 0.014335 | 0.116087 | 0.079762 | CXCL10/SOD2 |
| BP | GO:0006816 | calcium ion transport | 0.01443 | 0.116087 | 0.079762 | CXCL10/CXCL11/HSPA2 |
| BP | GO:0001967 | suckling behavior | 0.014587 | 0.116087 | 0.079762 | DACH1 |
| BP | GO:0010269 | response to selenium ion | 0.014587 | 0.116087 | 0.079762 | SOD2 |
| BP | GO:0015780 | nucleotide-sugar transmembrane transport | 0.014587 | 0.116087 | 0.079762 | SLC35D3 |
| BP | GO:0090677 | reversible differentiation | 0.014587 | 0.116087 | 0.079762 | SOD2 |
| BP | GO:1901142 | insulin metabolic process | 0.014587 | 0.116087 | 0.079762 | CPE |
| BP | GO:1901503 | ether biosynthetic process | 0.014587 | 0.116087 | 0.079762 | PLA2G4A |
| BP | GO:0097553 | calcium ion transmembrane import into cytosol | 0.015068 | 0.116087 | 0.079762 | CXCL10/CXCL11 |
| BP | GO:0007189 | adenylate cyclase-activating G protein-coupled receptor signaling pathway | 0.015254 | 0.116087 | 0.079762 | CXCL10/CXCL11 |
| BP | GO:0090316 | positive regulation of intracellular protein transport | 0.015441 | 0.116087 | 0.079762 | CHP2/SLC35D3 |
| BP | GO:0120254 | olefinic compound metabolic process | 0.015628 | 0.116087 | 0.079762 | ADH1C/PLA2G4A |
| BP | GO:0009314 | response to radiation | 0.015735 | 0.116087 | 0.079762 | CXCL10/MMP1/SOD2 |
| BP | GO:0002664 | regulation of T cell tolerance induction | 0.015793 | 0.116087 | 0.079762 | CLC |
| BP | GO:0033262 | regulation of nuclear cell cycle DNA replication | 0.015793 | 0.116087 | 0.079762 | DACH1 |
| BP | GO:0070486 | leukocyte aggregation | 0.015793 | 0.116087 | 0.079762 | S100A8 |
| BP | GO:0071361 | cellular response to ethanol | 0.015793 | 0.116087 | 0.079762 | SOD2 |
| BP | GO:1901894 | regulation of ATPase-coupled calcium transmembrane transporter activity | 0.015793 | 0.116087 | 0.079762 | HSPA2 |
| BP | GO:0035966 | response to topologically incorrect protein | 0.016197 | 0.116087 | 0.079762 | HSPA2/HSPA4L |
| BP | GO:0001976 | nervous system process involved in regulation of systemic arterial blood pressure | 0.016998 | 0.116087 | 0.079762 | SOD2 |
| BP | GO:0032306 | regulation of prostaglandin secretion | 0.016998 | 0.116087 | 0.079762 | PLA2G4A |
| BP | GO:0032308 | positive regulation of prostaglandin secretion | 0.016998 | 0.116087 | 0.079762 | PLA2G4A |
| BP | GO:0036151 | phosphatidylcholine acyl-chain remodeling | 0.016998 | 0.116087 | 0.079762 | PLA2G4A |
| BP | GO:0046462 | monoacylglycerol metabolic process | 0.016998 | 0.116087 | 0.079762 | PLA2G4A |
| BP | GO:0070141 | response to UV-A | 0.016998 | 0.116087 | 0.079762 | MMP1 |
| BP | GO:0071236 | cellular response to antibiotic | 0.016998 | 0.116087 | 0.079762 | PLA2G4A |
| BP | GO:1905065 | positive regulation of vascular associated smooth muscle cell differentiation | 0.016998 | 0.116087 | 0.079762 | SOD2 |
| BP | GO:0002468 | dendritic cell antigen processing and presentation | 0.018201 | 0.116087 | 0.079762 | CLEC4A |
| BP | GO:0002517 | T cell tolerance induction | 0.018201 | 0.116087 | 0.079762 | CLC |
| BP | GO:0003214 | cardiac left ventricle morphogenesis | 0.018201 | 0.116087 | 0.079762 | CPE |
| BP | GO:0070863 | positive regulation of protein exit from endoplasmic reticulum | 0.018201 | 0.116087 | 0.079762 | SLC35D3 |
| BP | GO:1905288 | vascular associated smooth muscle cell apoptotic process | 0.018201 | 0.116087 | 0.079762 | SOD2 |
| BP | GO:1905459 | regulation of vascular associated smooth muscle cell apoptotic process | 0.018201 | 0.116087 | 0.079762 | SOD2 |
| BP | GO:0060402 | calcium ion transport into cytosol | 0.018359 | 0.116087 | 0.079762 | CXCL10/CXCL11 |
| BP | GO:2001242 | regulation of intrinsic apoptotic signaling pathway | 0.018359 | 0.116087 | 0.079762 | S100A8/SOD2 |
| BP | GO:0002822 | regulation of adaptive immune response based on somatic recombination of immune receptors built from immunoglobulin superfamily domains | 0.018764 | 0.116087 | 0.079762 | CLC/PLA2G4A |
| BP | GO:0042129 | regulation of T cell proliferation | 0.018968 | 0.116087 | 0.079762 | CLC/TNFSF9 |
| BP | GO:0017014 | protein nitrosylation | 0.019404 | 0.116087 | 0.079762 | S100A8 |
| BP | GO:0018119 | peptidyl-cysteine S-nitrosylation | 0.019404 | 0.116087 | 0.079762 | S100A8 |
| BP | GO:0034393 | positive regulation of smooth muscle cell apoptotic process | 0.019404 | 0.116087 | 0.079762 | SOD2 |
| BP | GO:0034638 | phosphatidylcholine catabolic process | 0.019404 | 0.116087 | 0.079762 | PLA2G4A |
| BP | GO:0051238 | sequestering of metal ion | 0.019404 | 0.116087 | 0.079762 | S100A8 |
| BP | GO:0070886 | positive regulation of calcineurin-NFAT signaling cascade | 0.019404 | 0.116087 | 0.079762 | CHP2 |
| BP | GO:0106058 | positive regulation of calcineurin-mediated signaling | 0.019404 | 0.116087 | 0.079762 | CHP2 |
| BP | GO:0034765 | regulation of ion transmembrane transport | 0.019608 | 0.116255 | 0.079878 | CXCL10/CXCL11/HSPA2 |
| BP | GO:0002544 | chronic inflammatory response | 0.020604 | 0.116255 | 0.079878 | S100A8 |
| BP | GO:0010819 | regulation of T cell chemotaxis | 0.020604 | 0.116255 | 0.079878 | CXCL10 |
| BP | GO:0032310 | prostaglandin secretion | 0.020604 | 0.116255 | 0.079878 | PLA2G4A |
| BP | GO:0036166 | phenotypic switching | 0.020604 | 0.116255 | 0.079878 | SOD2 |
| BP | GO:0090083 | regulation of inclusion body assembly | 0.020604 | 0.116255 | 0.079878 | HSPA2 |
| BP | GO:0140374 | antiviral innate immune response | 0.020604 | 0.116255 | 0.079878 | CXCL10 |
| BP | GO:0072503 | cellular divalent inorganic cation homeostasis | 0.021612 | 0.117311 | 0.080604 | CXCL10/CXCL11/S100A8 |
| BP | GO:0002827 | positive regulation of T-helper 1 type immune response | 0.021804 | 0.117311 | 0.080604 | PLA2G4A |
| BP | GO:0003159 | morphogenesis of an endothelium | 0.021804 | 0.117311 | 0.080604 | CXCL10 |
| BP | GO:0050665 | hydrogen peroxide biosynthetic process | 0.021804 | 0.117311 | 0.080604 | SOD2 |
| BP | GO:0061154 | endothelial tube morphogenesis | 0.021804 | 0.117311 | 0.080604 | CXCL10 |
| BP | GO:0002819 | regulation of adaptive immune response | 0.021926 | 0.117311 | 0.080604 | CLC/PLA2G4A |
| BP | GO:0060401 | cytosolic calcium ion transport | 0.022363 | 0.117311 | 0.080604 | CXCL10/CXCL11 |
| BP | GO:0002523 | leukocyte migration involved in inflammatory response | 0.023001 | 0.117311 | 0.080604 | S100A8 |
| BP | GO:0006071 | glycerol metabolic process | 0.023001 | 0.117311 | 0.080604 | PLA2G4A |
| BP | GO:0015732 | prostaglandin transport | 0.023001 | 0.117311 | 0.080604 | PLA2G4A |
| BP | GO:0051152 | positive regulation of smooth muscle cell differentiation | 0.023001 | 0.117311 | 0.080604 | SOD2 |
| BP | GO:0055093 | response to hyperoxia | 0.023001 | 0.117311 | 0.080604 | SOD2 |
| BP | GO:0032388 | positive regulation of intracellular transport | 0.024148 | 0.117311 | 0.080604 | CHP2/SLC35D3 |
| BP | GO:0002643 | regulation of tolerance induction | 0.024198 | 0.117311 | 0.080604 | CLC |
| BP | GO:0010310 | regulation of hydrogen peroxide metabolic process | 0.024198 | 0.117311 | 0.080604 | SOD2 |
| BP | GO:0035902 | response to immobilization stress | 0.024198 | 0.117311 | 0.080604 | SOD2 |
| BP | GO:0046485 | ether lipid metabolic process | 0.024198 | 0.117311 | 0.080604 | PLA2G4A |
| BP | GO:0090026 | positive regulation of monocyte chemotaxis | 0.024198 | 0.117311 | 0.080604 | CXCL10 |
| BP | GO:1902176 | negative regulation of oxidative stress-induced intrinsic apoptotic signaling pathway | 0.024198 | 0.117311 | 0.080604 | SOD2 |
| BP | GO:0071674 | mononuclear cell migration | 0.024375 | 0.117311 | 0.080604 | CXCL10/CXCL11 |
| BP | GO:0003085 | negative regulation of systemic arterial blood pressure | 0.025393 | 0.117311 | 0.080604 | SOD2 |
| BP | GO:0006067 | ethanol metabolic process | 0.025393 | 0.117311 | 0.080604 | ADH1C |
| BP | GO:0019370 | leukotriene biosynthetic process | 0.025393 | 0.117311 | 0.080604 | PLA2G4A |
| BP | GO:0019400 | alditol metabolic process | 0.025393 | 0.117311 | 0.080604 | PLA2G4A |
| BP | GO:0032305 | positive regulation of icosanoid secretion | 0.025393 | 0.117311 | 0.080604 | PLA2G4A |
| BP | GO:0061760 | antifungal innate immune response | 0.025393 | 0.117311 | 0.080604 | CLEC4A |
| BP | GO:1901739 | regulation of myoblast fusion | 0.025393 | 0.117311 | 0.080604 | CXCL10 |
| BP | GO:0042098 | T cell proliferation | 0.025524 | 0.117311 | 0.080604 | CLC/TNFSF9 |
| BP | GO:0006662 | glycerol ether metabolic process | 0.026587 | 0.119039 | 0.081791 | PLA2G4A |
| BP | GO:0010042 | response to manganese ion | 0.026587 | 0.119039 | 0.081791 | SOD2 |
| BP | GO:0032303 | regulation of icosanoid secretion | 0.026587 | 0.119039 | 0.081791 | PLA2G4A |
| BP | GO:1903428 | positive regulation of reactive oxygen species biosynthetic process | 0.026587 | 0.119039 | 0.081791 | SOD2 |
| BP | GO:0006457 | protein folding | 0.027409 | 0.121934 | 0.08378 | HSPA2/HSPA4L |
| BP | GO:0019430 | removal of superoxide radicals | 0.027779 | 0.122793 | 0.08437 | SOD2 |
| BP | GO:0007601 | visual perception | 0.028615 | 0.124876 | 0.085801 | HMCN1/ZIC2 |
| BP | GO:0071222 | cellular response to lipopolysaccharide | 0.028615 | 0.124876 | 0.085801 | CXCL10/CXCL11 |
| BP | GO:0042026 | protein refolding | 0.02897 | 0.124876 | 0.085801 | HSPA2 |
| BP | GO:0070841 | inclusion body assembly | 0.02897 | 0.124876 | 0.085801 | HSPA2 |
| BP | GO:0050953 | sensory perception of light stimulus | 0.029594 | 0.125745 | 0.086399 | HMCN1/ZIC2 |
| BP | GO:0045662 | negative regulation of myoblast differentiation | 0.030159 | 0.125745 | 0.086399 | CXCL10 |
| BP | GO:0071450 | cellular response to oxygen radical | 0.030159 | 0.125745 | 0.086399 | SOD2 |
| BP | GO:0071451 | cellular response to superoxide | 0.030159 | 0.125745 | 0.086399 | SOD2 |
| BP | GO:2000193 | positive regulation of fatty acid transport | 0.030159 | 0.125745 | 0.086399 | PLA2G4A |
| BP | GO:0033157 | regulation of intracellular protein transport | 0.030338 | 0.125745 | 0.086399 | CHP2/SLC35D3 |
| BP | GO:0002790 | peptide secretion | 0.030588 | 0.125745 | 0.086399 | CPE/S100A8 |
| BP | GO:0036296 | response to increased oxygen levels | 0.031347 | 0.125745 | 0.086399 | SOD2 |
| BP | GO:0070193 | synaptonemal complex organization | 0.031347 | 0.125745 | 0.086399 | HSPA2 |
| BP | GO:1901623 | regulation of lymphocyte chemotaxis | 0.031347 | 0.125745 | 0.086399 | CXCL10 |
| BP | GO:1905063 | regulation of vascular associated smooth muscle cell differentiation | 0.031347 | 0.125745 | 0.086399 | SOD2 |
| BP | GO:0071219 | cellular response to molecule of bacterial origin | 0.031594 | 0.125745 | 0.086399 | CXCL10/CXCL11 |
| BP | GO:0042445 | hormone metabolic process | 0.031848 | 0.125745 | 0.086399 | ADH1C/CPE |
| BP | GO:0050670 | regulation of lymphocyte proliferation | 0.031848 | 0.125745 | 0.086399 | CLC/TNFSF9 |
| BP | GO:0010996 | response to auditory stimulus | 0.032534 | 0.125745 | 0.086399 | CXCL10 |
| BP | GO:0018904 | ether metabolic process | 0.032534 | 0.125745 | 0.086399 | PLA2G4A |
| BP | GO:0036037 | CD8-positive, alpha-beta T cell activation | 0.032534 | 0.125745 | 0.086399 | CLEC4A |
| BP | GO:0070861 | regulation of protein exit from endoplasmic reticulum | 0.032534 | 0.125745 | 0.086399 | SLC35D3 |
| BP | GO:0032944 | regulation of mononuclear cell proliferation | 0.032614 | 0.125745 | 0.086399 | CLC/TNFSF9 |
| BP | GO:0097305 | response to alcohol | 0.033129 | 0.125813 | 0.086445 | S100A8/SOD2 |
| BP | GO:0000303 | response to superoxide | 0.033719 | 0.125813 | 0.086445 | SOD2 |
| BP | GO:0007141 | male meiosis I | 0.033719 | 0.125813 | 0.086445 | HSPA2 |
| BP | GO:0010971 | positive regulation of G2/M transition of mitotic cell cycle | 0.033719 | 0.125813 | 0.086445 | HSPA2 |
| BP | GO:0042104 | positive regulation of activated T cell proliferation | 0.033719 | 0.125813 | 0.086445 | TNFSF9 |
| BP | GO:0090025 | regulation of monocyte chemotaxis | 0.033719 | 0.125813 | 0.086445 | CXCL10 |
| BP | GO:0000305 | response to oxygen radical | 0.034903 | 0.127489 | 0.087597 | SOD2 |
| BP | GO:0002474 | antigen processing and presentation of peptide antigen via MHC class I | 0.034903 | 0.127489 | 0.087597 | CLEC4A |
| BP | GO:0002825 | regulation of T-helper 1 type immune response | 0.034903 | 0.127489 | 0.087597 | PLA2G4A |
| BP | GO:1902175 | regulation of oxidative stress-induced intrinsic apoptotic signaling pathway | 0.034903 | 0.127489 | 0.087597 | SOD2 |
| BP | GO:0007188 | adenylate cyclase-modulating G protein-coupled receptor signaling pathway | 0.035485 | 0.128673 | 0.08841 | CXCL10/CXCL11 |
| BP | GO:0043032 | positive regulation of macrophage activation | 0.036086 | 0.128673 | 0.08841 | PLA2G4A |
| BP | GO:0060142 | regulation of syncytium formation by plasma membrane fusion | 0.036086 | 0.128673 | 0.08841 | CXCL10 |
| BP | GO:0015833 | peptide transport | 0.036822 | 0.128673 | 0.08841 | CPE/S100A8 |
| BP | GO:0002507 | tolerance induction | 0.037267 | 0.128673 | 0.08841 | CLC |
| BP | GO:0034390 | smooth muscle cell apoptotic process | 0.037267 | 0.128673 | 0.08841 | SOD2 |
| BP | GO:0034391 | regulation of smooth muscle cell apoptotic process | 0.037267 | 0.128673 | 0.08841 | SOD2 |
| BP | GO:0050482 | arachidonic acid secretion | 0.037267 | 0.128673 | 0.08841 | PLA2G4A |
| BP | GO:1902751 | positive regulation of cell cycle G2/M phase transition | 0.037267 | 0.128673 | 0.08841 | HSPA2 |
| BP | GO:1903963 | arachidonate transport | 0.037267 | 0.128673 | 0.08841 | PLA2G4A |
| BP | GO:2000406 | positive regulation of T cell migration | 0.037267 | 0.128673 | 0.08841 | CXCL10 |
| BP | GO:0070663 | regulation of leukocyte proliferation | 0.038178 | 0.129524 | 0.088995 | CLC/TNFSF9 |
| BP | GO:0001516 | prostaglandin biosynthetic process | 0.038447 | 0.129524 | 0.088995 | PLA2G4A |
| BP | GO:0046457 | prostanoid biosynthetic process | 0.038447 | 0.129524 | 0.088995 | PLA2G4A |
| BP | GO:0051085 | chaperone cofactor-dependent protein refolding | 0.038447 | 0.129524 | 0.088995 | HSPA2 |
| BP | GO:0071711 | basement membrane organization | 0.038447 | 0.129524 | 0.088995 | HMCN1 |
| BP | GO:0071216 | cellular response to biotic stimulus | 0.038726 | 0.129835 | 0.089208 | CXCL10/CXCL11 |
| BP | GO:0010661 | positive regulation of muscle cell apoptotic process | 0.039625 | 0.131578 | 0.090406 | SOD2 |
| BP | GO:2000191 | regulation of fatty acid transport | 0.039625 | 0.131578 | 0.090406 | PLA2G4A |
| BP | GO:0006691 | leukotriene metabolic process | 0.040802 | 0.132321 | 0.090917 | PLA2G4A |
| BP | GO:0016486 | peptide hormone processing | 0.040802 | 0.132321 | 0.090917 | CPE |
| BP | GO:0042573 | retinoic acid metabolic process | 0.040802 | 0.132321 | 0.090917 | ADH1C |
| BP | GO:0046475 | glycerophospholipid catabolic process | 0.040802 | 0.132321 | 0.090917 | PLA2G4A |
| BP | GO:0050850 | positive regulation of calcium-mediated signaling | 0.040802 | 0.132321 | 0.090917 | CHP2 |
| BP | GO:0051091 | positive regulation of DNA-binding transcription factor activity | 0.041229 | 0.133083 | 0.09144 | S100A8/ZIC2 |
| BP | GO:0033280 | response to vitamin D | 0.041978 | 0.134251 | 0.092243 | CXCL10 |
| BP | GO:0070884 | regulation of calcineurin-NFAT signaling cascade | 0.041978 | 0.134251 | 0.092243 | CHP2 |
| BP | GO:0010922 | positive regulation of phosphatase activity | 0.043152 | 0.134898 | 0.092688 | CHP2 |
| BP | GO:0046471 | phosphatidylglycerol metabolic process | 0.043152 | 0.134898 | 0.092688 | PLA2G4A |
| BP | GO:0050931 | pigment cell differentiation | 0.043152 | 0.134898 | 0.092688 | SOD2 |
| BP | GO:0106056 | regulation of calcineurin-mediated signaling | 0.043152 | 0.134898 | 0.092688 | CHP2 |
| BP | GO:0140448 | signaling receptor ligand precursor processing | 0.043152 | 0.134898 | 0.092688 | CPE |
| BP | GO:0051084 | 'de novo' post-translational protein folding | 0.044325 | 0.136096 | 0.093511 | HSPA2 |
| BP | GO:1901021 | positive regulation of calcium ion transmembrane transporter activity | 0.044325 | 0.136096 | 0.093511 | HSPA2 |
| BP | GO:2000279 | negative regulation of DNA biosynthetic process | 0.044325 | 0.136096 | 0.093511 | DACH1 |
| BP | GO:2000403 | positive regulation of lymphocyte migration | 0.044325 | 0.136096 | 0.093511 | CXCL10 |
| BP | GO:0046942 | carboxylic acid transport | 0.045242 | 0.136096 | 0.093511 | PLA2G4A/SLC35D3 |
| BP | GO:0002369 | T cell cytokine production | 0.045496 | 0.136096 | 0.093511 | CLC |
| BP | GO:0002478 | antigen processing and presentation of exogenous peptide antigen | 0.045496 | 0.136096 | 0.093511 | CLEC4A |
| BP | GO:0002724 | regulation of T cell cytokine production | 0.045496 | 0.136096 | 0.093511 | CLC |
| BP | GO:0033260 | nuclear DNA replication | 0.045496 | 0.136096 | 0.093511 | DACH1 |
| BP | GO:0035886 | vascular associated smooth muscle cell differentiation | 0.045496 | 0.136096 | 0.093511 | SOD2 |
| BP | GO:0006882 | cellular zinc ion homeostasis | 0.046666 | 0.137815 | 0.094691 | S100A8 |
| BP | GO:0009595 | detection of biotic stimulus | 0.046666 | 0.137815 | 0.094691 | DACH1 |
| BP | GO:0042307 | positive regulation of protein import into nucleus | 0.046666 | 0.137815 | 0.094691 | CHP2 |
| BP | GO:0042886 | amide transport | 0.047598 | 0.139972 | 0.096173 | CPE/S100A8 |
| BP | GO:0097009 | energy homeostasis | 0.047835 | 0.140074 | 0.096244 | SLC35D3 |
| BP | GO:0006458 | 'de novo' protein folding | 0.049002 | 0.140775 | 0.096726 | HSPA2 |
| BP | GO:0045429 | positive regulation of nitric oxide biosynthetic process | 0.049002 | 0.140775 | 0.096726 | SOD2 |
| BP | GO:0055069 | zinc ion homeostasis | 0.049002 | 0.140775 | 0.096726 | S100A8 |
| BP | GO:0051222 | positive regulation of protein transport | 0.049395 | 0.140775 | 0.096726 | CHP2/SLC35D3 |
| BP | GO:0030574 | collagen catabolic process | 0.050168 | 0.140775 | 0.096726 | MMP1 |
| BP | GO:0051602 | response to electrical stimulus | 0.050168 | 0.140775 | 0.096726 | SOD2 |
| BP | GO:1904591 | positive regulation of protein import | 0.050168 | 0.140775 | 0.096726 | CHP2 |
| BP | GO:1904706 | negative regulation of vascular associated smooth muscle cell proliferation | 0.050168 | 0.140775 | 0.096726 | SOD2 |
| BP | GO:0046651 | lymphocyte proliferation | 0.050303 | 0.140775 | 0.096726 | CLC/TNFSF9 |
| BP | GO:0014002 | astrocyte development | 0.051333 | 0.140775 | 0.096726 | S100A8 |
| BP | GO:0042554 | superoxide anion generation | 0.051333 | 0.140775 | 0.096726 | SOD2 |
| BP | GO:0044786 | cell cycle DNA replication | 0.051333 | 0.140775 | 0.096726 | DACH1 |
| BP | GO:0051150 | regulation of smooth muscle cell differentiation | 0.051333 | 0.140775 | 0.096726 | SOD2 |
| BP | GO:0140353 | lipid export from cell | 0.051333 | 0.140775 | 0.096726 | PLA2G4A |
| BP | GO:1904407 | positive regulation of nitric oxide metabolic process | 0.051333 | 0.140775 | 0.096726 | SOD2 |
| BP | GO:0032943 | mononuclear cell proliferation | 0.051523 | 0.140775 | 0.096726 | CLC/TNFSF9 |
| BP | GO:0051047 | positive regulation of secretion | 0.051523 | 0.140775 | 0.096726 | PLA2G4A/S100A8 |
| BP | GO:0033173 | calcineurin-NFAT signaling cascade | 0.052496 | 0.142123 | 0.097651 | CHP2 |
| BP | GO:2000404 | regulation of T cell migration | 0.052496 | 0.142123 | 0.097651 | CXCL10 |
| BP | GO:0007520 | myoblast fusion | 0.053658 | 0.142123 | 0.097651 | CXCL10 |
| BP | GO:0008631 | intrinsic apoptotic signaling pathway in response to oxidative stress | 0.053658 | 0.142123 | 0.097651 | SOD2 |
| BP | GO:0032781 | positive regulation of ATP-dependent activity | 0.053658 | 0.142123 | 0.097651 | HSPA2 |
| BP | GO:0032892 | positive regulation of organic acid transport | 0.053658 | 0.142123 | 0.097651 | PLA2G4A |
| BP | GO:0042088 | T-helper 1 type immune response | 0.053658 | 0.142123 | 0.097651 | PLA2G4A |
| BP | GO:0030198 | extracellular matrix organization | 0.053684 | 0.142123 | 0.097651 | HMCN1/MMP1 |
| BP | GO:0043062 | extracellular structure organization | 0.053995 | 0.142123 | 0.097651 | HMCN1/MMP1 |
| BP | GO:1904951 | positive regulation of establishment of protein localization | 0.054307 | 0.142123 | 0.097651 | CHP2/SLC35D3 |
| BP | GO:0045229 | external encapsulating structure organization | 0.05462 | 0.142123 | 0.097651 | HMCN1/MMP1 |
| BP | GO:0032309 | icosanoid secretion | 0.054819 | 0.142123 | 0.097651 | PLA2G4A |
| BP | GO:0046677 | response to antibiotic | 0.054819 | 0.142123 | 0.097651 | PLA2G4A |
| BP | GO:0019884 | antigen processing and presentation of exogenous antigen | 0.055978 | 0.142123 | 0.097651 | CLEC4A |
| BP | GO:0045776 | negative regulation of blood pressure | 0.055978 | 0.142123 | 0.097651 | SOD2 |
| BP | GO:0048066 | developmental pigmentation | 0.055978 | 0.142123 | 0.097651 | SOD2 |
| BP | GO:0006959 | humoral immune response | 0.056827 | 0.142123 | 0.097651 | CXCL10/CXCL11 |
| BP | GO:0003044 | regulation of systemic arterial blood pressure mediated by a chemical signal | 0.057136 | 0.142123 | 0.097651 | SOD2 |
| BP | GO:0010543 | regulation of platelet activation | 0.057136 | 0.142123 | 0.097651 | PLA2G4A |
| BP | GO:0032527 | protein exit from endoplasmic reticulum | 0.057136 | 0.142123 | 0.097651 | SLC35D3 |
| BP | GO:0042311 | vasodilation | 0.057136 | 0.142123 | 0.097651 | SOD2 |
| BP | GO:0046460 | neutral lipid biosynthetic process | 0.057136 | 0.142123 | 0.097651 | PLA2G4A |
| BP | GO:0046463 | acylglycerol biosynthetic process | 0.057136 | 0.142123 | 0.097651 | PLA2G4A |
| BP | GO:0097720 | calcineurin-mediated signaling | 0.057136 | 0.142123 | 0.097651 | CHP2 |
| BP | GO:1903426 | regulation of reactive oxygen species biosynthetic process | 0.057136 | 0.142123 | 0.097651 | SOD2 |
| BP | GO:0018198 | peptidyl-cysteine modification | 0.058292 | 0.143967 | 0.098919 | S100A8 |
| BP | GO:1903793 | positive regulation of anion transport | 0.058292 | 0.143967 | 0.098919 | PLA2G4A |
| BP | GO:0007204 | positive regulation of cytosolic calcium ion concentration | 0.059387 | 0.144759 | 0.099463 | CXCL10/CXCL11 |
| BP | GO:0006692 | prostanoid metabolic process | 0.059447 | 0.144759 | 0.099463 | PLA2G4A |
| BP | GO:0006693 | prostaglandin metabolic process | 0.059447 | 0.144759 | 0.099463 | PLA2G4A |
| BP | GO:0070231 | T cell apoptotic process | 0.059447 | 0.144759 | 0.099463 | CLC |
| BP | GO:0007140 | male meiotic nuclear division | 0.060601 | 0.146032 | 0.100337 | HSPA2 |
| BP | GO:0007157 | heterophilic cell-cell adhesion via plasma membrane cell adhesion molecules | 0.060601 | 0.146032 | 0.100337 | HMCN1 |
| BP | GO:0042572 | retinol metabolic process | 0.060601 | 0.146032 | 0.100337 | ADH1C |
| BP | GO:0070661 | leukocyte proliferation | 0.061008 | 0.146269 | 0.1005 | CLC/TNFSF9 |
| BP | GO:0032386 | regulation of intracellular transport | 0.061661 | 0.146269 | 0.1005 | CHP2/SLC35D3 |
| BP | GO:0006636 | unsaturated fatty acid biosynthetic process | 0.061753 | 0.146269 | 0.1005 | PLA2G4A |
| BP | GO:0009395 | phospholipid catabolic process | 0.061753 | 0.146269 | 0.1005 | PLA2G4A |
| BP | GO:0090329 | regulation of DNA-templated DNA replication | 0.061753 | 0.146269 | 0.1005 | DACH1 |
| BP | GO:0045661 | regulation of myoblast differentiation | 0.064054 | 0.150181 | 0.103188 | CXCL10 |
| BP | GO:0071715 | icosanoid transport | 0.064054 | 0.150181 | 0.103188 | PLA2G4A |
| BP | GO:1903202 | negative regulation of oxidative stress-induced cell death | 0.064054 | 0.150181 | 0.103188 | SOD2 |
| BP | GO:0050863 | regulation of T cell activation | 0.064961 | 0.151793 | 0.104296 | CLC/TNFSF9 |
| BP | GO:0042743 | hydrogen peroxide metabolic process | 0.065203 | 0.151847 | 0.104333 | SOD2 |
| BP | GO:0001836 | release of cytochrome c from mitochondria | 0.06635 | 0.153489 | 0.105461 | SOD2 |
| BP | GO:0048016 | inositol phosphate-mediated signaling | 0.06635 | 0.153489 | 0.105461 | CHP2 |
| BP | GO:0042306 | regulation of protein import into nucleus | 0.067495 | 0.154593 | 0.10622 | CHP2 |
| BP | GO:0046456 | icosanoid biosynthetic process | 0.067495 | 0.154593 | 0.10622 | PLA2G4A |
| BP | GO:0051289 | protein homotetramerization | 0.067495 | 0.154593 | 0.10622 | SOD2 |
| BP | GO:0019369 | arachidonic acid metabolic process | 0.068639 | 0.156697 | 0.107665 | PLA2G4A |
| BP | GO:0051480 | regulation of cytosolic calcium ion concentration | 0.06968 | 0.156728 | 0.107687 | CXCL10/CXCL11 |
| BP | GO:0000768 | syncytium formation by plasma membrane fusion | 0.069782 | 0.156728 | 0.107687 | CXCL10 |
| BP | GO:0045599 | negative regulation of fat cell differentiation | 0.069782 | 0.156728 | 0.107687 | SOD2 |
| BP | GO:0046686 | response to cadmium ion | 0.069782 | 0.156728 | 0.107687 | SOD2 |
| BP | GO:0140253 | cell-cell fusion | 0.069782 | 0.156728 | 0.107687 | CXCL10 |
| BP | GO:0046824 | positive regulation of nucleocytoplasmic transport | 0.070924 | 0.157769 | 0.108402 | CHP2 |
| BP | GO:0006066 | alcohol metabolic process | 0.071393 | 0.157769 | 0.108402 | ADH1C/PLA2G4A |
| BP | GO:0015711 | organic anion transport | 0.071393 | 0.157769 | 0.108402 | PLA2G4A/SLC35D3 |
| BP | GO:0035306 | positive regulation of dephosphorylation | 0.072064 | 0.157769 | 0.108402 | CHP2 |
| BP | GO:0045428 | regulation of nitric oxide biosynthetic process | 0.072064 | 0.157769 | 0.108402 | SOD2 |
| BP | GO:1903409 | reactive oxygen species biosynthetic process | 0.072064 | 0.157769 | 0.108402 | SOD2 |
| BP | GO:1904589 | regulation of protein import | 0.072064 | 0.157769 | 0.108402 | CHP2 |
| BP | GO:2001244 | positive regulation of intrinsic apoptotic signaling pathway | 0.072064 | 0.157769 | 0.108402 | S100A8 |
| BP | GO:0022617 | extracellular matrix disassembly | 0.073203 | 0.159111 | 0.109324 | MMP1 |
| BP | GO:0043030 | regulation of macrophage activation | 0.073203 | 0.159111 | 0.109324 | PLA2G4A |
| BP | GO:0006949 | syncytium formation | 0.074341 | 0.159111 | 0.109324 | CXCL10 |
| BP | GO:0048002 | antigen processing and presentation of peptide antigen | 0.074341 | 0.159111 | 0.109324 | CLEC4A |
| BP | GO:0051965 | positive regulation of synapse assembly | 0.074341 | 0.159111 | 0.109324 | LRRN1 |
| BP | GO:2000401 | regulation of lymphocyte migration | 0.074341 | 0.159111 | 0.109324 | CXCL10 |
| BP | GO:0002460 | adaptive immune response based on somatic recombination of immune receptors built from immunoglobulin superfamily domains | 0.074512 | 0.159111 | 0.109324 | CLC/PLA2G4A |
| BP | GO:2001233 | regulation of apoptotic signaling pathway | 0.074512 | 0.159111 | 0.109324 | S100A8/SOD2 |
| BP | GO:0050918 | positive chemotaxis | 0.075477 | 0.160187 | 0.110063 | CXCL10 |
| BP | GO:0080164 | regulation of nitric oxide metabolic process | 0.075477 | 0.160187 | 0.110063 | SOD2 |
| BP | GO:0006749 | glutathione metabolic process | 0.076612 | 0.161061 | 0.110664 | SOD2 |
| BP | GO:0014823 | response to activity | 0.076612 | 0.161061 | 0.110664 | SOD2 |
| BP | GO:1905330 | regulation of morphogenesis of an epithelium | 0.076612 | 0.161061 | 0.110664 | CXCL10 |
| BP | GO:0015909 | long-chain fatty acid transport | 0.077746 | 0.161061 | 0.110664 | PLA2G4A |
| BP | GO:0034605 | cellular response to heat | 0.077746 | 0.161061 | 0.110664 | CXCL10 |
| BP | GO:0038034 | signal transduction in absence of ligand | 0.077746 | 0.161061 | 0.110664 | BCL2A1 |
| BP | GO:0071677 | positive regulation of mononuclear cell migration | 0.077746 | 0.161061 | 0.110664 | CXCL10 |
| BP | GO:0097192 | extrinsic apoptotic signaling pathway in absence of ligand | 0.077746 | 0.161061 | 0.110664 | BCL2A1 |
| BP | GO:0007159 | leukocyte cell-cell adhesion | 0.078383 | 0.161899 | 0.111239 | S100A8/TNFSF9 |
| BP | GO:0046503 | glycerolipid catabolic process | 0.080009 | 0.164766 | 0.113209 | PLA2G4A |
| BP | GO:0042692 | muscle cell differentiation | 0.080522 | 0.165332 | 0.113599 | CXCL10/SOD2 |
| BP | GO:0032370 | positive regulation of lipid transport | 0.081138 | 0.166106 | 0.11413 | PLA2G4A |
| BP | GO:0002548 | monocyte chemotaxis | 0.082267 | 0.166452 | 0.114368 | CXCL10 |
| BP | GO:0007585 | respiratory gaseous exchange by respiratory system | 0.082267 | 0.166452 | 0.114368 | DACH1 |
| BP | GO:0032890 | regulation of organic acid transport | 0.082267 | 0.166452 | 0.114368 | PLA2G4A |
| BP | GO:0061077 | chaperone-mediated protein folding | 0.082267 | 0.166452 | 0.114368 | HSPA2 |
| BP | GO:0051881 | regulation of mitochondrial membrane potential | 0.083394 | 0.167754 | 0.115263 | SOD2 |
| BP | GO:2000379 | positive regulation of reactive oxygen species metabolic process | 0.083394 | 0.167754 | 0.115263 | SOD2 |
| BP | GO:0003208 | cardiac ventricle morphogenesis | 0.084519 | 0.169039 | 0.116145 | CPE |
| BP | GO:0006801 | superoxide metabolic process | 0.084519 | 0.169039 | 0.116145 | SOD2 |
| BP | GO:0043462 | regulation of ATP-dependent activity | 0.085644 | 0.169819 | 0.116681 | HSPA2 |
| BP | GO:0048662 | negative regulation of smooth muscle cell proliferation | 0.085644 | 0.169819 | 0.116681 | SOD2 |
| BP | GO:0051145 | smooth muscle cell differentiation | 0.085644 | 0.169819 | 0.116681 | SOD2 |
| BP | GO:0046470 | phosphatidylcholine metabolic process | 0.086767 | 0.171556 | 0.117875 | PLA2G4A |
| BP | GO:0006809 | nitric oxide biosynthetic process | 0.087888 | 0.172301 | 0.118387 | SOD2 |
| BP | GO:0050848 | regulation of calcium-mediated signaling | 0.087888 | 0.172301 | 0.118387 | CHP2 |
| BP | GO:0070227 | lymphocyte apoptotic process | 0.087888 | 0.172301 | 0.118387 | CLC |
| BP | GO:0009410 | response to xenobiotic stimulus | 0.08926 | 0.17423 | 0.119712 | CD69/SOD2 |
| BP | GO:0006919 | activation of cysteine-type endopeptidase activity involved in apoptotic process | 0.090128 | 0.17423 | 0.119712 | S100A8 |
| BP | GO:0032720 | negative regulation of tumor necrosis factor production | 0.090128 | 0.17423 | 0.119712 | CLEC4A |
| BP | GO:0051149 | positive regulation of muscle cell differentiation | 0.090128 | 0.17423 | 0.119712 | SOD2 |
| BP | GO:1903201 | regulation of oxidative stress-induced cell death | 0.090128 | 0.17423 | 0.119712 | SOD2 |
| BP | GO:1901264 | carbohydrate derivative transport | 0.091245 | 0.175901 | 0.12086 | SLC35D3 |
| BP | GO:0048708 | astrocyte differentiation | 0.092362 | 0.177069 | 0.121663 | S100A8 |
| BP | GO:1903556 | negative regulation of tumor necrosis factor superfamily cytokine production | 0.092362 | 0.177069 | 0.121663 | CLEC4A |
| BP | GO:0002696 | positive regulation of leukocyte activation | 0.092984 | 0.177771 | 0.122145 | PLA2G4A/TNFSF9 |
| BP | GO:0010660 | regulation of muscle cell apoptotic process | 0.094591 | 0.17936 | 0.123237 | SOD2 |
| BP | GO:0045445 | myoblast differentiation | 0.094591 | 0.17936 | 0.123237 | CXCL10 |
| BP | GO:0046209 | nitric oxide metabolic process | 0.094591 | 0.17936 | 0.123237 | SOD2 |
| BP | GO:2001057 | reactive nitrogen species metabolic process | 0.095703 | 0.180975 | 0.124347 | SOD2 |
| BP | GO:0042060 | wound healing | 0.095996 | 0.181036 | 0.124389 | PLA2G4A/S100A8 |
| BP | GO:0048678 | response to axon injury | 0.096814 | 0.182084 | 0.125109 | SOD2 |
| BP | GO:0001523 | retinoid metabolic process | 0.097924 | 0.182197 | 0.125186 | ADH1C |
| BP | GO:0010921 | regulation of phosphatase activity | 0.097924 | 0.182197 | 0.125186 | CHP2 |
| BP | GO:0071277 | cellular response to calcium ion | 0.097924 | 0.182197 | 0.125186 | CHP2 |
| BP | GO:1900182 | positive regulation of protein localization to nucleus | 0.097924 | 0.182197 | 0.125186 | CHP2 |
| BP | GO:0050867 | positive regulation of cell activation | 0.098656 | 0.182304 | 0.12526 | PLA2G4A/TNFSF9 |
| BP | GO:0010657 | muscle cell apoptotic process | 0.099033 | 0.182304 | 0.12526 | SOD2 |
| BP | GO:0055072 | iron ion homeostasis | 0.099033 | 0.182304 | 0.12526 | SOD2 |
| BP | GO:0098586 | cellular response to virus | 0.099033 | 0.182304 | 0.12526 | CXCL10 |
| BP | GO:1904705 | regulation of vascular associated smooth muscle cell proliferation | 0.10014 | 0.183797 | 0.126285 | SOD2 |
| BP | GO:0032103 | positive regulation of response to external stimulus | 0.100952 | 0.183797 | 0.126285 | CXCL10/S100A8 |
| BP | GO:0009791 | post-embryonic development | 0.101246 | 0.183797 | 0.126285 | SOD2 |
| BP | GO:0016101 | diterpenoid metabolic process | 0.101246 | 0.183797 | 0.126285 | ADH1C |
| BP | GO:1990874 | vascular associated smooth muscle cell proliferation | 0.101246 | 0.183797 | 0.126285 | SOD2 |
| BP | GO:0002709 | regulation of T cell mediated immunity | 0.102351 | 0.183797 | 0.126285 | CLC |
| BP | GO:0034644 | cellular response to UV | 0.102351 | 0.183797 | 0.126285 | MMP1 |
| BP | GO:0051262 | protein tetramerization | 0.102351 | 0.183797 | 0.126285 | SOD2 |
| BP | GO:0031667 | response to nutrient levels | 0.102492 | 0.183797 | 0.126285 | CXCL10/SOD2 |
| BP | GO:1903829 | positive regulation of protein localization | 0.102492 | 0.183797 | 0.126285 | CHP2/SLC35D3 |
| BP | GO:0044070 | regulation of anion transport | 0.103454 | 0.185044 | 0.127142 | PLA2G4A |
| BP | GO:0023061 | signal release | 0.104426 | 0.186303 | 0.128007 | CPE/PLA2G4A |
| BP | GO:0051090 | regulation of DNA-binding transcription factor activity | 0.104814 | 0.186515 | 0.128153 | S100A8/ZIC2 |
| BP | GO:0097306 | cellular response to alcohol | 0.105657 | 0.187056 | 0.128524 | SOD2 |
| BP | GO:1901019 | regulation of calcium ion transmembrane transporter activity | 0.105657 | 0.187056 | 0.128524 | HSPA2 |
| BP | GO:0006874 | cellular calcium ion homeostasis | 0.106371 | 0.18784 | 0.129064 | CXCL10/CXCL11 |
| BP | GO:1900407 | regulation of cellular response to oxidative stress | 0.106756 | 0.188043 | 0.129203 | SOD2 |
| BP | GO:0002793 | positive regulation of peptide secretion | 0.107854 | 0.189157 | 0.129968 | S100A8 |
| BP | GO:0022411 | cellular component disassembly | 0.107934 | 0.189157 | 0.129968 | HSPA2/MMP1 |
| BP | GO:0002690 | positive regulation of leukocyte chemotaxis | 0.108951 | 0.189504 | 0.130207 | CXCL10 |
| BP | GO:0045582 | positive regulation of T cell differentiation | 0.108951 | 0.189504 | 0.130207 | TNFSF9 |
| BP | GO:1905954 | positive regulation of lipid localization | 0.108951 | 0.189504 | 0.130207 | PLA2G4A |
| BP | GO:0003073 | regulation of systemic arterial blood pressure | 0.110047 | 0.190931 | 0.131187 | SOD2 |
| BP | GO:0055074 | calcium ion homeostasis | 0.111079 | 0.192242 | 0.132088 | CXCL10/CXCL11 |
| BP | GO:0010389 | regulation of G2/M transition of mitotic cell cycle | 0.112234 | 0.193715 | 0.1331 | HSPA2 |
| BP | GO:0002718 | regulation of cytokine production involved in immune response | 0.113326 | 0.193715 | 0.1331 | CLC |
| BP | GO:0006721 | terpenoid metabolic process | 0.113326 | 0.193715 | 0.1331 | ADH1C |
| BP | GO:0034620 | cellular response to unfolded protein | 0.113326 | 0.193715 | 0.1331 | HSPA2 |
| BP | GO:0036473 | cell death in response to oxidative stress | 0.113326 | 0.193715 | 0.1331 | SOD2 |
| BP | GO:0015908 | fatty acid transport | 0.114416 | 0.195038 | 0.134009 | PLA2G4A |
| BP | GO:0009991 | response to extracellular stimulus | 0.115445 | 0.195038 | 0.134009 | CXCL10/SOD2 |
| BP | GO:0002367 | cytokine production involved in immune response | 0.115505 | 0.195038 | 0.134009 | CLC |
| BP | GO:0098869 | cellular oxidant detoxification | 0.115505 | 0.195038 | 0.134009 | SOD2 |
| BP | GO:1901992 | positive regulation of mitotic cell cycle phase transition | 0.115505 | 0.195038 | 0.134009 | HSPA2 |
| BP | GO:0032963 | collagen metabolic process | 0.116593 | 0.195449 | 0.134291 | MMP1 |
| BP | GO:0043473 | pigmentation | 0.116593 | 0.195449 | 0.134291 | SOD2 |
| BP | GO:1902882 | regulation of response to oxidative stress | 0.116593 | 0.195449 | 0.134291 | SOD2 |
| BP | GO:2001243 | negative regulation of intrinsic apoptotic signaling pathway | 0.11768 | 0.196795 | 0.135216 | SOD2 |
| BP | GO:0019221 | cytokine-mediated signaling pathway | 0.118247 | 0.197184 | 0.135484 | CXCL10/CXCL11 |
| BP | GO:0042102 | positive regulation of T cell proliferation | 0.118765 | 0.197184 | 0.135484 | TNFSF9 |
| BP | GO:0051963 | regulation of synapse assembly | 0.118765 | 0.197184 | 0.135484 | LRRN1 |
| BP | GO:0046822 | regulation of nucleocytoplasmic transport | 0.119849 | 0.198207 | 0.136186 | CHP2 |
| BP | GO:0034308 | primary alcohol metabolic process | 0.120932 | 0.198207 | 0.136186 | ADH1C |
| BP | GO:1901890 | positive regulation of cell junction assembly | 0.120932 | 0.198207 | 0.136186 | LRRN1 |
| BP | GO:0006091 | generation of precursor metabolites and energy | 0.121471 | 0.198207 | 0.136186 | ADH1C/SOD2 |
| BP | GO:0044089 | positive regulation of cellular component biogenesis | 0.121471 | 0.198207 | 0.136186 | LRRN1/MMP1 |
| BP | GO:0051223 | regulation of protein transport | 0.121876 | 0.198207 | 0.136186 | CHP2/SLC35D3 |
| BP | GO:0007631 | feeding behavior | 0.122014 | 0.198207 | 0.136186 | DACH1 |
| BP | GO:0032414 | positive regulation of ion transmembrane transporter activity | 0.122014 | 0.198207 | 0.136186 | HSPA2 |
| BP | GO:0042116 | macrophage activation | 0.122014 | 0.198207 | 0.136186 | PLA2G4A |
| BP | GO:0010817 | regulation of hormone levels | 0.122281 | 0.198207 | 0.136186 | ADH1C/CPE |
| BP | GO:0008637 | apoptotic mitochondrial changes | 0.123094 | 0.198207 | 0.136186 | SOD2 |
| BP | GO:0019882 | antigen processing and presentation | 0.123094 | 0.198207 | 0.136186 | CLEC4A |
| BP | GO:0045621 | positive regulation of lymphocyte differentiation | 0.123094 | 0.198207 | 0.136186 | TNFSF9 |
| BP | GO:0007218 | neuropeptide signaling pathway | 0.124173 | 0.199021 | 0.136746 | CPE |
| BP | GO:1902749 | regulation of cell cycle G2/M phase transition | 0.124173 | 0.199021 | 0.136746 | HSPA2 |
| BP | GO:0046545 | development of primary female sexual characteristics | 0.12525 | 0.199825 | 0.137298 | DACH1 |
| BP | GO:0071887 | leukocyte apoptotic process | 0.12525 | 0.199825 | 0.137298 | CLC |
| BP | GO:0002824 | positive regulation of adaptive immune response based on somatic recombination of immune receptors built from immunoglobulin superfamily domains | 0.126327 | 0.20108 | 0.13816 | PLA2G4A |
| BP | GO:0001676 | long-chain fatty acid metabolic process | 0.127402 | 0.201865 | 0.1387 | PLA2G4A |
| BP | GO:0031532 | actin cytoskeleton reorganization | 0.127402 | 0.201865 | 0.1387 | HMCN1 |
| BP | GO:0002456 | T cell mediated immunity | 0.128476 | 0.203103 | 0.139551 | CLC |
| BP | GO:0002526 | acute inflammatory response | 0.129549 | 0.20387 | 0.140078 | S100A8 |
| BP | GO:0022904 | respiratory electron transport chain | 0.129549 | 0.20387 | 0.140078 | SOD2 |
| BP | GO:0002821 | positive regulation of adaptive immune response | 0.13169 | 0.204917 | 0.140797 | PLA2G4A |
| BP | GO:0021782 | glial cell development | 0.13169 | 0.204917 | 0.140797 | S100A8 |
| BP | GO:0033559 | unsaturated fatty acid metabolic process | 0.13169 | 0.204917 | 0.140797 | PLA2G4A |
| BP | GO:0046916 | cellular transition metal ion homeostasis | 0.13169 | 0.204917 | 0.140797 | S100A8 |
| BP | GO:1990748 | cellular detoxification | 0.13169 | 0.204917 | 0.140797 | SOD2 |
| BP | GO:0019751 | polyol metabolic process | 0.132759 | 0.205477 | 0.141182 | PLA2G4A |
| BP | GO:0071675 | regulation of mononuclear cell migration | 0.132759 | 0.205477 | 0.141182 | CXCL10 |
| BP | GO:0006720 | isoprenoid metabolic process | 0.133826 | 0.205477 | 0.141182 | ADH1C |
| BP | GO:0015718 | monocarboxylic acid transport | 0.133826 | 0.205477 | 0.141182 | PLA2G4A |
| BP | GO:0032411 | positive regulation of transporter activity | 0.133826 | 0.205477 | 0.141182 | HSPA2 |
| BP | GO:0035967 | cellular response to topologically incorrect protein | 0.133826 | 0.205477 | 0.141182 | HSPA2 |
| BP | GO:0032368 | regulation of lipid transport | 0.134893 | 0.206657 | 0.141992 | PLA2G4A |
| BP | GO:2000278 | regulation of DNA biosynthetic process | 0.135958 | 0.20783 | 0.142798 | DACH1 |
| BP | GO:0006690 | icosanoid metabolic process | 0.138084 | 0.210154 | 0.144395 | PLA2G4A |
| BP | GO:0071482 | cellular response to light stimulus | 0.138084 | 0.210154 | 0.144395 | MMP1 |
| BP | GO:0014902 | myotube differentiation | 0.139145 | 0.211306 | 0.145187 | CXCL10 |
| BP | GO:0097237 | cellular response to toxic substance | 0.140205 | 0.211988 | 0.145655 | SOD2 |
| BP | GO:1901989 | positive regulation of cell cycle phase transition | 0.140205 | 0.211988 | 0.145655 | HSPA2 |
| BP | GO:0002688 | regulation of leukocyte chemotaxis | 0.141264 | 0.212662 | 0.146119 | CXCL10 |
| BP | GO:0046660 | female sex differentiation | 0.141264 | 0.212662 | 0.146119 | DACH1 |
| BP | GO:0043280 | positive regulation of cysteine-type endopeptidase activity involved in apoptotic process | 0.142322 | 0.213791 | 0.146894 | S100A8 |
| BP | GO:0003206 | cardiac chamber morphogenesis | 0.143378 | 0.214449 | 0.147346 | CPE |
| BP | GO:0003231 | cardiac ventricle development | 0.143378 | 0.214449 | 0.147346 | CPE |
| BP | GO:0042542 | response to hydrogen peroxide | 0.145487 | 0.21667 | 0.148872 | SOD2 |
| BP | GO:0045931 | positive regulation of mitotic cell cycle | 0.145487 | 0.21667 | 0.148872 | HSPA2 |
| BP | GO:0007127 | meiosis I | 0.147591 | 0.218397 | 0.150059 | HSPA2 |
| BP | GO:0030168 | platelet activation | 0.147591 | 0.218397 | 0.150059 | PLA2G4A |
| BP | GO:0035303 | regulation of dephosphorylation | 0.147591 | 0.218397 | 0.150059 | CHP2 |
| BP | GO:0006275 | regulation of DNA replication | 0.148641 | 0.219017 | 0.150485 | DACH1 |
| BP | GO:0006639 | acylglycerol metabolic process | 0.148641 | 0.219017 | 0.150485 | PLA2G4A |
| BP | GO:0006638 | neutral lipid metabolic process | 0.14969 | 0.21963 | 0.150906 | PLA2G4A |
| BP | GO:1900180 | regulation of protein localization to nucleus | 0.14969 | 0.21963 | 0.150906 | CHP2 |
| BP | GO:0003158 | endothelium development | 0.150738 | 0.2207 | 0.151641 | CXCL10 |
| BP | GO:0001889 | liver development | 0.151784 | 0.221298 | 0.152052 | SOD2 |
| BP | GO:2001235 | positive regulation of apoptotic signaling pathway | 0.151784 | 0.221298 | 0.152052 | S100A8 |
| BP | GO:0061982 | meiosis I cell cycle process | 0.153873 | 0.223874 | 0.153822 | HSPA2 |
| BP | GO:0034754 | cellular hormone metabolic process | 0.154916 | 0.22445 | 0.154218 | ADH1C |
| BP | GO:0061008 | hepaticobiliary system development | 0.154916 | 0.22445 | 0.154218 | SOD2 |
| BP | GO:0000086 | G2/M transition of mitotic cell cycle | 0.158036 | 0.227624 | 0.156399 | HSPA2 |
| BP | GO:0050921 | positive regulation of chemotaxis | 0.158036 | 0.227624 | 0.156399 | CXCL10 |
| BP | GO:0002687 | positive regulation of leukocyte migration | 0.159074 | 0.227624 | 0.156399 | CXCL10 |
| BP | GO:0035296 | regulation of tube diameter | 0.159074 | 0.227624 | 0.156399 | SOD2 |
| BP | GO:0050671 | positive regulation of lymphocyte proliferation | 0.159074 | 0.227624 | 0.156399 | TNFSF9 |
| BP | GO:0097746 | blood vessel diameter maintenance | 0.159074 | 0.227624 | 0.156399 | SOD2 |
| BP | GO:0035150 | regulation of tube size | 0.160111 | 0.228166 | 0.156771 | SOD2 |
| BP | GO:0045598 | regulation of fat cell differentiation | 0.160111 | 0.228166 | 0.156771 | SOD2 |
| BP | GO:0032946 | positive regulation of mononuclear cell proliferation | 0.161146 | 0.228702 | 0.15714 | TNFSF9 |
| BP | GO:2001056 | positive regulation of cysteine-type endopeptidase activity | 0.161146 | 0.228702 | 0.15714 | S100A8 |
| BP | GO:0051592 | response to calcium ion | 0.16218 | 0.2297 | 0.157825 | CHP2 |
| BP | GO:0043524 | negative regulation of neuron apoptotic process | 0.163213 | 0.230223 | 0.158185 | SOD2 |
| BP | GO:0050729 | positive regulation of inflammatory response | 0.163213 | 0.230223 | 0.158185 | S100A8 |
| BP | GO:0009411 | response to UV | 0.164245 | 0.231209 | 0.158862 | MMP1 |
| BP | GO:0034614 | cellular response to reactive oxygen species | 0.165275 | 0.231719 | 0.159213 | SOD2 |
| BP | GO:2000377 | regulation of reactive oxygen species metabolic process | 0.165275 | 0.231719 | 0.159213 | SOD2 |
| BP | GO:0051053 | negative regulation of DNA metabolic process | 0.166305 | 0.232692 | 0.159881 | DACH1 |
| BP | GO:0016525 | negative regulation of angiogenesis | 0.167333 | 0.23366 | 0.160546 | CXCL10 |
| BP | GO:0098754 | detoxification | 0.168359 | 0.234621 | 0.161207 | SOD2 |
| BP | GO:0045580 | regulation of T cell differentiation | 0.169385 | 0.235106 | 0.16154 | TNFSF9 |
| BP | GO:2000181 | negative regulation of blood vessel morphogenesis | 0.169385 | 0.235106 | 0.16154 | CXCL10 |
| BP | GO:1901343 | negative regulation of vasculature development | 0.170409 | 0.236056 | 0.162192 | CXCL10 |
| BP | GO:0051147 | regulation of muscle cell differentiation | 0.171433 | 0.237001 | 0.162841 | SOD2 |
| BP | GO:0051092 | positive regulation of NF-kappaB transcription factor activity | 0.172455 | 0.237939 | 0.163486 | S100A8 |
| BP | GO:0044839 | cell cycle G2/M phase transition | 0.173475 | 0.2384 | 0.163803 | HSPA2 |
| BP | GO:1905952 | regulation of lipid localization | 0.173475 | 0.2384 | 0.163803 | PLA2G4A |
| BP | GO:0008643 | carbohydrate transport | 0.174495 | 0.239305 | 0.164425 | SLC35D3 |
| BP | GO:0046434 | organophosphate catabolic process | 0.175513 | 0.239305 | 0.164425 | PLA2G4A |
| BP | GO:1902107 | positive regulation of leukocyte differentiation | 0.175513 | 0.239305 | 0.164425 | TNFSF9 |
| BP | GO:1903708 | positive regulation of hemopoiesis | 0.175513 | 0.239305 | 0.164425 | TNFSF9 |
| BP | GO:0046631 | alpha-beta T cell activation | 0.176531 | 0.23975 | 0.16473 | CLEC4A |
| BP | GO:0070665 | positive regulation of leukocyte proliferation | 0.176531 | 0.23975 | 0.16473 | TNFSF9 |
| BP | GO:0006261 | DNA-templated DNA replication | 0.177547 | 0.24019 | 0.165033 | DACH1 |
| BP | GO:0006606 | protein import into nucleus | 0.177547 | 0.24019 | 0.165033 | CHP2 |
| BP | GO:0006633 | fatty acid biosynthetic process | 0.180587 | 0.24377 | 0.167493 | PLA2G4A |
| BP | GO:0007568 | aging | 0.181598 | 0.24377 | 0.167493 | SOD2 |
| BP | GO:0030307 | positive regulation of cell growth | 0.181598 | 0.24377 | 0.167493 | S100A8 |
| BP | GO:0051170 | import into nucleus | 0.181598 | 0.24377 | 0.167493 | CHP2 |
| BP | GO:0002700 | regulation of production of molecular mediator of immune response | 0.185631 | 0.247746 | 0.170225 | CLC |
| BP | GO:0003205 | cardiac chamber development | 0.185631 | 0.247746 | 0.170225 | CPE |
| BP | GO:0022900 | electron transport chain | 0.185631 | 0.247746 | 0.170225 | SOD2 |
| BP | GO:0007156 | homophilic cell adhesion via plasma membrane adhesion molecules | 0.186637 | 0.248134 | 0.170491 | HMCN1 |
| BP | GO:0071466 | cellular response to xenobiotic stimulus | 0.186637 | 0.248134 | 0.170491 | CD69 |
| BP | GO:0048660 | regulation of smooth muscle cell proliferation | 0.188643 | 0.250322 | 0.171995 | SOD2 |
| BP | GO:0048659 | smooth muscle cell proliferation | 0.191645 | 0.25382 | 0.174398 | SOD2 |
| BP | GO:0010950 | positive regulation of endopeptidase activity | 0.192643 | 0.254656 | 0.174972 | S100A8 |
| BP | GO:0002706 | regulation of lymphocyte mediated immunity | 0.19364 | 0.255487 | 0.175543 | CLC |
| BP | GO:0007286 | spermatid development | 0.19563 | 0.257623 | 0.177011 | HSPA2 |
| BP | GO:0002791 | regulation of peptide secretion | 0.198607 | 0.260555 | 0.179025 | S100A8 |
| BP | GO:0007416 | synapse assembly | 0.198607 | 0.260555 | 0.179025 | LRRN1 |
| BP | GO:0045619 | regulation of lymphocyte differentiation | 0.199597 | 0.261359 | 0.179578 | TNFSF9 |
| BP | GO:0032640 | tumor necrosis factor production | 0.200586 | 0.26148 | 0.179661 | CLEC4A |
| BP | GO:0032680 | regulation of tumor necrosis factor production | 0.200586 | 0.26148 | 0.179661 | CLEC4A |
| BP | GO:0008217 | regulation of blood pressure | 0.201573 | 0.26148 | 0.179661 | SOD2 |
| BP | GO:0048515 | spermatid differentiation | 0.201573 | 0.26148 | 0.179661 | HSPA2 |
| BP | GO:0090087 | regulation of peptide transport | 0.201573 | 0.26148 | 0.179661 | S100A8 |
| BP | GO:0051260 | protein homooligomerization | 0.205512 | 0.264611 | 0.181812 | SOD2 |
| BP | GO:0071478 | cellular response to radiation | 0.205512 | 0.264611 | 0.181812 | MMP1 |
| BP | GO:0071706 | tumor necrosis factor superfamily cytokine production | 0.205512 | 0.264611 | 0.181812 | CLEC4A |
| BP | GO:1903555 | regulation of tumor necrosis factor superfamily cytokine production | 0.205512 | 0.264611 | 0.181812 | CLEC4A |
| BP | GO:0006575 | cellular modified amino acid metabolic process | 0.206494 | 0.264892 | 0.182005 | SOD2 |
| BP | GO:0140013 | meiotic nuclear division | 0.206494 | 0.264892 | 0.182005 | HSPA2 |
| BP | GO:0071248 | cellular response to metal ion | 0.209432 | 0.268166 | 0.184255 | CHP2 |
| BP | GO:0007626 | locomotory behavior | 0.210409 | 0.268426 | 0.184434 | SOD2 |
| BP | GO:0010952 | positive regulation of peptidase activity | 0.210409 | 0.268426 | 0.184434 | S100A8 |
| BP | GO:0031334 | positive regulation of protein-containing complex assembly | 0.21236 | 0.269923 | 0.185462 | MMP1 |
| BP | GO:0071897 | DNA biosynthetic process | 0.21236 | 0.269923 | 0.185462 | DACH1 |
| BP | GO:0034284 | response to monosaccharide | 0.218184 | 0.276819 | 0.1902 | SOD2 |
| BP | GO:0009612 | response to mechanical stimulus | 0.219151 | 0.277033 | 0.190347 | CXCL10 |
| BP | GO:1901888 | regulation of cell junction assembly | 0.219151 | 0.277033 | 0.190347 | LRRN1 |
| BP | GO:0019722 | calcium-mediated signaling | 0.220117 | 0.277747 | 0.190838 | CHP2 |
| BP | GO:0000302 | response to reactive oxygen species | 0.221081 | 0.278458 | 0.191326 | SOD2 |
| BP | GO:0043281 | regulation of cysteine-type endopeptidase activity involved in apoptotic process | 0.222045 | 0.279165 | 0.191812 | S100A8 |
| BP | GO:0043523 | regulation of neuron apoptotic process | 0.224928 | 0.281769 | 0.193601 | SOD2 |
| BP | GO:1903046 | meiotic cell cycle process | 0.224928 | 0.281769 | 0.193601 | HSPA2 |
| BP | GO:0050807 | regulation of synapse organization | 0.226844 | 0.283147 | 0.194548 | LRRN1 |
| BP | GO:1901215 | negative regulation of neuron death | 0.226844 | 0.283147 | 0.194548 | SOD2 |
| BP | GO:0017038 | protein import | 0.227801 | 0.283831 | 0.195018 | CHP2 |
| BP | GO:0046474 | glycerophospholipid biosynthetic process | 0.231616 | 0.288067 | 0.197928 | PLA2G4A |
| BP | GO:0050803 | regulation of synapse structure or activity | 0.232566 | 0.288216 | 0.198031 | LRRN1 |
| BP | GO:0072330 | monocarboxylic acid biosynthetic process | 0.232566 | 0.288216 | 0.198031 | PLA2G4A |
| BP | GO:0002685 | regulation of leukocyte migration | 0.235412 | 0.290188 | 0.199386 | CXCL10 |
| BP | GO:0010001 | glial cell differentiation | 0.235412 | 0.290188 | 0.199386 | S100A8 |
| BP | GO:0015931 | nucleobase-containing compound transport | 0.235412 | 0.290188 | 0.199386 | SLC35D3 |
| BP | GO:0044242 | cellular lipid catabolic process | 0.236359 | 0.290838 | 0.199833 | PLA2G4A |
| BP | GO:0030072 | peptide hormone secretion | 0.237304 | 0.291099 | 0.200012 | CPE |
| BP | GO:0007596 | blood coagulation | 0.238248 | 0.291099 | 0.200012 | PLA2G4A |
| BP | GO:0071241 | cellular response to inorganic substance | 0.238248 | 0.291099 | 0.200012 | CHP2 |
| BP | GO:0097191 | extrinsic apoptotic signaling pathway | 0.238248 | 0.291099 | 0.200012 | BCL2A1 |
| BP | GO:0050870 | positive regulation of T cell activation | 0.240133 | 0.292886 | 0.20124 | TNFSF9 |
| BP | GO:0050920 | regulation of chemotaxis | 0.242013 | 0.294662 | 0.20246 | CXCL10 |
| BP | GO:0009743 | response to carbohydrate | 0.242952 | 0.29477 | 0.202534 | SOD2 |
| BP | GO:0050817 | coagulation | 0.242952 | 0.29477 | 0.202534 | PLA2G4A |
| BP | GO:0007599 | hemostasis | 0.243889 | 0.295391 | 0.202961 | PLA2G4A |
| BP | GO:2000116 | regulation of cysteine-type endopeptidase activity | 0.245761 | 0.29714 | 0.204162 | S100A8 |
| BP | GO:2001234 | negative regulation of apoptotic signaling pathway | 0.246695 | 0.297325 | 0.20429 | SOD2 |
| BP | GO:0045137 | development of primary sexual characteristics | 0.247628 | 0.297325 | 0.20429 | DACH1 |
| BP | GO:0045333 | cellular respiration | 0.247628 | 0.297325 | 0.20429 | SOD2 |
| BP | GO:0072593 | reactive oxygen species metabolic process | 0.247628 | 0.297325 | 0.20429 | SOD2 |
| BP | GO:0002274 | myeloid leukocyte activation | 0.24856 | 0.297929 | 0.204704 | PLA2G4A |
| BP | GO:0033002 | muscle cell proliferation | 0.249491 | 0.298529 | 0.205117 | SOD2 |
| BP | GO:0002703 | regulation of leukocyte mediated immunity | 0.252277 | 0.301342 | 0.20705 | CLC |
| BP | GO:0045444 | fat cell differentiation | 0.253203 | 0.301929 | 0.207453 | SOD2 |
| BP | GO:0051402 | neuron apoptotic process | 0.256898 | 0.305809 | 0.210119 | SOD2 |
| BP | GO:0016485 | protein processing | 0.258738 | 0.307473 | 0.211263 | CPE |
| BP | GO:0051259 | protein complex oligomerization | 0.259657 | 0.308038 | 0.21165 | SOD2 |
| BP | GO:1903039 | positive regulation of leukocyte cell-cell adhesion | 0.260575 | 0.308599 | 0.212036 | TNFSF9 |
| BP | GO:0009636 | response to toxic substance | 0.262407 | 0.309186 | 0.212439 | SOD2 |
| BP | GO:0090068 | positive regulation of cell cycle process | 0.262407 | 0.309186 | 0.212439 | HSPA2 |
| BP | GO:0098656 | anion transmembrane transport | 0.262407 | 0.309186 | 0.212439 | SLC35D3 |
| BP | GO:0045017 | glycerolipid biosynthetic process | 0.267877 | 0.315096 | 0.2165 | PLA2G4A |
| BP | GO:0003007 | heart morphogenesis | 0.268785 | 0.315629 | 0.216866 | CPE |
| BP | GO:0045927 | positive regulation of growth | 0.270597 | 0.317221 | 0.21796 | S100A8 |
| BP | GO:0008654 | phospholipid biosynthetic process | 0.272406 | 0.318802 | 0.219046 | PLA2G4A |
| BP | GO:0003018 | vascular process in circulatory system | 0.276908 | 0.322439 | 0.221546 | SOD2 |
| BP | GO:0030217 | T cell differentiation | 0.276908 | 0.322439 | 0.221546 | TNFSF9 |
| BP | GO:0032412 | regulation of ion transmembrane transporter activity | 0.276908 | 0.322439 | 0.221546 | HSPA2 |
| BP | GO:0051321 | meiotic cell cycle | 0.279596 | 0.325024 | 0.223322 | HSPA2 |
| BP | GO:0051962 | positive regulation of nervous system development | 0.283166 | 0.328624 | 0.225795 | LRRN1 |
| BP | GO:0022898 | regulation of transmembrane transporter activity | 0.285832 | 0.331164 | 0.22754 | HSPA2 |
| BP | GO:1903532 | positive regulation of secretion by cell | 0.286718 | 0.331637 | 0.227866 | PLA2G4A |
| BP | GO:0098742 | cell-cell adhesion via plasma-membrane adhesion molecules | 0.291135 | 0.335982 | 0.230851 | HMCN1 |
| BP | GO:0006260 | DNA replication | 0.292016 | 0.335982 | 0.230851 | DACH1 |
| BP | GO:0007548 | sex differentiation | 0.292895 | 0.335982 | 0.230851 | DACH1 |
| BP | GO:0046879 | hormone secretion | 0.292895 | 0.335982 | 0.230851 | CPE |
| BP | GO:0051146 | striated muscle cell differentiation | 0.292895 | 0.335982 | 0.230851 | CXCL10 |
| BP | GO:1902105 | regulation of leukocyte differentiation | 0.29465 | 0.337438 | 0.231851 | TNFSF9 |
| BP | GO:0034599 | cellular response to oxidative stress | 0.295526 | 0.337884 | 0.232157 | SOD2 |
| BP | GO:0001666 | response to hypoxia | 0.297276 | 0.339324 | 0.233147 | SOD2 |
| BP | GO:0044262 | cellular carbohydrate metabolic process | 0.298149 | 0.339762 | 0.233448 | PLA2G4A |
| BP | GO:0031349 | positive regulation of defense response | 0.299891 | 0.340376 | 0.23387 | S100A8 |
| BP | GO:0009914 | hormone transport | 0.300761 | 0.340376 | 0.23387 | CPE |
| BP | GO:0051607 | defense response to virus | 0.300761 | 0.340376 | 0.23387 | CXCL10 |
| BP | GO:0022409 | positive regulation of cell-cell adhesion | 0.30163 | 0.340376 | 0.23387 | TNFSF9 |
| BP | GO:0042063 | gliogenesis | 0.30163 | 0.340376 | 0.23387 | S100A8 |
| BP | GO:0140546 | defense response to symbiont | 0.30163 | 0.340376 | 0.23387 | CXCL10 |
| BP | GO:0034504 | protein localization to nucleus | 0.304231 | 0.342753 | 0.235503 | CHP2 |
| BP | GO:0007281 | germ cell development | 0.308544 | 0.346488 | 0.238069 | HSPA2 |
| BP | GO:0036293 | response to decreased oxygen levels | 0.308544 | 0.346488 | 0.238069 | SOD2 |
| BP | GO:0019932 | second-messenger-mediated signaling | 0.311976 | 0.349776 | 0.240329 | CHP2 |
| BP | GO:0032409 | regulation of transporter activity | 0.313686 | 0.351126 | 0.241256 | HSPA2 |
| BP | GO:0006913 | nucleocytoplasmic transport | 0.316244 | 0.352851 | 0.242441 | CHP2 |
| BP | GO:0051169 | nuclear transport | 0.316244 | 0.352851 | 0.242441 | CHP2 |
| BP | GO:0006650 | glycerophospholipid metabolic process | 0.317094 | 0.353232 | 0.242703 | PLA2G4A |
| BP | GO:0009416 | response to light stimulus | 0.317943 | 0.35361 | 0.242963 | MMP1 |
| BP | GO:0002440 | production of molecular mediator of immune response | 0.319639 | 0.354928 | 0.243868 | CLC |
| BP | GO:1901214 | regulation of neuron death | 0.320486 | 0.355299 | 0.244123 | SOD2 |
| BP | GO:0051604 | protein maturation | 0.321331 | 0.355668 | 0.244377 | CPE |
| BP | GO:0046394 | carboxylic acid biosynthetic process | 0.323019 | 0.356967 | 0.245269 | PLA2G4A |
| BP | GO:0015849 | organic acid transport | 0.324703 | 0.357688 | 0.245765 | PLA2G4A |
| BP | GO:0016053 | organic acid biosynthetic process | 0.324703 | 0.357688 | 0.245765 | PLA2G4A |
| BP | GO:0015980 | energy derivation by oxidation of organic compounds | 0.327221 | 0.359322 | 0.246887 | SOD2 |
| BP | GO:1901990 | regulation of mitotic cell cycle phase transition | 0.327221 | 0.359322 | 0.246887 | HSPA2 |
| BP | GO:0071214 | cellular response to abiotic stimulus | 0.328895 | 0.36002 | 0.247367 | MMP1 |
| BP | GO:0104004 | cellular response to environmental stimulus | 0.328895 | 0.36002 | 0.247367 | MMP1 |
| BP | GO:0070482 | response to oxygen levels | 0.32973 | 0.360366 | 0.247605 | SOD2 |
| BP | GO:0016042 | lipid catabolic process | 0.33223 | 0.362528 | 0.24909 | PLA2G4A |
| BP | GO:0060562 | epithelial tube morphogenesis | 0.333062 | 0.362865 | 0.249321 | CXCL10 |
| BP | GO:0045787 | positive regulation of cell cycle | 0.333892 | 0.363199 | 0.249551 | HSPA2 |
| BP | GO:0062197 | cellular response to chemical stress | 0.336377 | 0.36533 | 0.251015 | SOD2 |
| BP | GO:0007517 | muscle organ development | 0.338029 | 0.36655 | 0.251854 | CXCL10 |
| BP | GO:0006790 | sulfur compound metabolic process | 0.342142 | 0.370431 | 0.254521 | SOD2 |
| BP | GO:1903037 | regulation of leukocyte cell-cell adhesion | 0.34623 | 0.374274 | 0.257161 | TNFSF9 |
| BP | GO:0045765 | regulation of angiogenesis | 0.347045 | 0.374571 | 0.257365 | CXCL10 |
| BP | GO:0030336 | negative regulation of cell migration | 0.347859 | 0.374866 | 0.257568 | DACH1 |
| BP | GO:1901342 | regulation of vasculature development | 0.351913 | 0.378648 | 0.260166 | CXCL10 |
| BP | GO:0002697 | regulation of immune effector process | 0.353528 | 0.379209 | 0.260552 | CLC |
| BP | GO:0070997 | neuron death | 0.353528 | 0.379209 | 0.260552 | SOD2 |
| BP | GO:2000146 | negative regulation of cell motility | 0.359949 | 0.385501 | 0.264875 | DACH1 |
| BP | GO:0001818 | negative regulation of cytokine production | 0.361545 | 0.386614 | 0.265639 | CLEC4A |
| BP | GO:0042742 | defense response to bacterium | 0.362342 | 0.386869 | 0.265815 | S100A8 |
| BP | GO:0002449 | lymphocyte mediated immunity | 0.363137 | 0.387123 | 0.265989 | CLC |
| BP | GO:0045862 | positive regulation of proteolysis | 0.36631 | 0.389309 | 0.267491 | S100A8 |
| BP | GO:0051271 | negative regulation of cellular component movement | 0.36631 | 0.389309 | 0.267491 | DACH1 |
| BP | GO:0051251 | positive regulation of lymphocyte activation | 0.36789 | 0.389795 | 0.267825 | TNFSF9 |
| BP | GO:1903706 | regulation of hemopoiesis | 0.36789 | 0.389795 | 0.267825 | TNFSF9 |
| BP | GO:0030098 | lymphocyte differentiation | 0.376517 | 0.397721 | 0.273271 | TNFSF9 |
| BP | GO:0050878 | regulation of body fluid levels | 0.376517 | 0.397721 | 0.273271 | PLA2G4A |
| BP | GO:0006644 | phospholipid metabolic process | 0.381174 | 0.402029 | 0.276231 | PLA2G4A |
| BP | GO:0006869 | lipid transport | 0.38349 | 0.403858 | 0.277488 | PLA2G4A |
| BP | GO:0009615 | response to virus | 0.384261 | 0.404056 | 0.277624 | CXCL10 |
| BP | GO:0050727 | regulation of inflammatory response | 0.385799 | 0.404833 | 0.278157 | S100A8 |
| BP | GO:0006631 | fatty acid metabolic process | 0.386566 | 0.404833 | 0.278157 | PLA2G4A |
| BP | GO:0040013 | negative regulation of locomotion | 0.387333 | 0.404833 | 0.278157 | DACH1 |
| BP | GO:0046486 | glycerolipid metabolic process | 0.387333 | 0.404833 | 0.278157 | PLA2G4A |
| BP | GO:0043254 | regulation of protein-containing complex assembly | 0.391152 | 0.40821 | 0.280478 | MMP1 |
| BP | GO:0022412 | cellular process involved in reproduction in multicellular organism | 0.394948 | 0.411553 | 0.282775 | HSPA2 |
| BP | GO:0001558 | regulation of cell growth | 0.401725 | 0.417361 | 0.286765 | S100A8 |
| BP | GO:1901987 | regulation of cell cycle phase transition | 0.401725 | 0.417361 | 0.286765 | HSPA2 |
| BP | GO:0050808 | synapse organization | 0.404713 | 0.419837 | 0.288467 | LRRN1 |
| BP | GO:0034329 | cell junction assembly | 0.405458 | 0.419982 | 0.288566 | LRRN1 |
| BP | GO:0016311 | dephosphorylation | 0.406945 | 0.420894 | 0.289193 | CHP2 |
| BP | GO:0042391 | regulation of membrane potential | 0.409169 | 0.422565 | 0.290341 | SOD2 |
| BP | GO:0052548 | regulation of endopeptidase activity | 0.409909 | 0.422699 | 0.290433 | S100A8 |
| BP | GO:0048732 | gland development | 0.413593 | 0.425235 | 0.292176 | SOD2 |
| BP | GO:0072594 | establishment of protein localization to organelle | 0.413593 | 0.425235 | 0.292176 | CHP2 |
| BP | GO:0006979 | response to oxidative stress | 0.415061 | 0.425483 | 0.292346 | SOD2 |
| BP | GO:1903131 | mononuclear cell differentiation | 0.415061 | 0.425483 | 0.292346 | TNFSF9 |
| BP | GO:0044772 | mitotic cell cycle phase transition | 0.42017 | 0.429452 | 0.295073 | HSPA2 |
| BP | GO:0051960 | regulation of nervous system development | 0.42017 | 0.429452 | 0.295073 | LRRN1 |
| BP | GO:0050673 | epithelial cell proliferation | 0.422346 | 0.431042 | 0.296165 | RETNLB |
| BP | GO:0000280 | nuclear division | 0.424515 | 0.431352 | 0.296379 | HSPA2 |
| BP | GO:0010876 | lipid localization | 0.424515 | 0.431352 | 0.296379 | PLA2G4A |
| BP | GO:0045785 | positive regulation of cell adhesion | 0.424515 | 0.431352 | 0.296379 | TNFSF9 |
| BP | GO:0007015 | actin filament organization | 0.427394 | 0.433643 | 0.297953 | HMCN1 |
| BP | GO:0016055 | Wnt signaling pathway | 0.428829 | 0.434463 | 0.298516 | CPE |
| BP | GO:0198738 | cell-cell signaling by wnt | 0.43026 | 0.435278 | 0.299076 | CPE |
| BP | GO:0022407 | regulation of cell-cell adhesion | 0.431688 | 0.435452 | 0.299196 | TNFSF9 |
| BP | GO:0052547 | regulation of peptidase activity | 0.431688 | 0.435452 | 0.299196 | S100A8 |
| BP | GO:0002443 | leukocyte mediated immunity | 0.4324 | 0.435538 | 0.299255 | CLC |
| BP | GO:0051052 | regulation of DNA metabolic process | 0.442987 | 0.445555 | 0.306137 | DACH1 |
| BP | GO:0007346 | regulation of mitotic cell cycle | 0.447168 | 0.44911 | 0.30858 | HSPA2 |
| BP | GO:0016049 | cell growth | 0.45132 | 0.452624 | 0.310994 | S100A8 |
| BP | GO:0090066 | regulation of anatomical structure size | 0.453384 | 0.454038 | 0.311966 | SOD2 |
| BP | GO:0048285 | organelle fission | 0.457491 | 0.457491 | 0.314338 | HSPA2 |
| CC | GO:0062023 | collagen-containing extracellular matrix | 0.013333 | 0.160165 | 0.137229 | CLC/HMCN1/S100A8 |
| CC | GO:0036128 | CatSper complex | 0.015157 | 0.160165 | 0.137229 | HSPA2 |
| CC | GO:0009897 | external side of plasma membrane | 0.015601 | 0.160165 | 0.137229 | CD69/CLEC4A/CXCL10 |
| CC | GO:0072687 | meiotic spindle | 0.016314 | 0.160165 | 0.137229 | HSPA2 |
| CC | GO:0001673 | male germ cell nucleus | 0.018624 | 0.160165 | 0.137229 | HSPA2 |
| CC | GO:0043073 | germ cell nucleus | 0.024375 | 0.174691 | 0.149674 | HSPA2 |
| CC | GO:0000795 | synaptonemal complex | 0.047061 | 0.197097 | 0.168871 | HSPA2 |
| CC | GO:0099086 | synaptonemal structure | 0.047061 | 0.197097 | 0.168871 | HSPA2 |
| CC | GO:0005891 | voltage-gated calcium channel complex | 0.05042 | 0.197097 | 0.168871 | HSPA2 |
| CC | GO:0009295 | nucleoid | 0.05042 | 0.197097 | 0.168871 | SOD2 |
| CC | GO:0042645 | mitochondrial nucleoid | 0.05042 | 0.197097 | 0.168871 | SOD2 |
| CC | GO:0032154 | cleavage furrow | 0.06264 | 0.22446 | 0.192315 | HMCN1 |
| CC | GO:0034704 | calcium channel complex | 0.076888 | 0.239061 | 0.204826 | HSPA2 |
| CC | GO:0032153 | cell division site | 0.082312 | 0.239061 | 0.204826 | HMCN1 |
| CC | GO:0000794 | condensed nuclear chromosome | 0.083393 | 0.239061 | 0.204826 | HSPA2 |
| CC | GO:0005604 | basement membrane | 0.10582 | 0.284391 | 0.243664 | HMCN1 |
| CC | GO:0072562 | blood microparticle | 0.159116 | 0.402469 | 0.344832 | HSPA2 |
| CC | GO:0005912 | adherens junction | 0.183644 | 0.431495 | 0.369702 | HMCN1 |
| CC | GO:0005741 | mitochondrial outer membrane | 0.214975 | 0.431495 | 0.369702 | BCL2A1 |
| CC | GO:0030658 | transport vesicle membrane | 0.214975 | 0.431495 | 0.369702 | CPE |
| CC | GO:0034703 | cation channel complex | 0.229748 | 0.431495 | 0.369702 | HSPA2 |
| CC | GO:0000228 | nuclear chromosome | 0.236127 | 0.431495 | 0.369702 | HSPA2 |
| CC | GO:0031968 | organelle outer membrane | 0.23975 | 0.431495 | 0.369702 | BCL2A1 |
| CC | GO:0019867 | outer membrane | 0.241555 | 0.431495 | 0.369702 | BCL2A1 |
| CC | GO:0000793 | condensed chromosome | 0.260264 | 0.431495 | 0.369702 | HSPA2 |
| CC | GO:0045111 | intermediate filament cytoskeleton | 0.262022 | 0.431495 | 0.369702 | S100A8 |
| CC | GO:0034702 | ion channel complex | 0.293843 | 0.431495 | 0.369702 | HSPA2 |
| CC | GO:0005938 | cell cortex | 0.307193 | 0.431495 | 0.369702 | HMCN1 |
| CC | GO:0030667 | secretory granule membrane | 0.308845 | 0.431495 | 0.369702 | CPE |
| CC | GO:0034774 | secretory granule lumen | 0.317047 | 0.431495 | 0.369702 | S100A8 |
| CC | GO:0060205 | cytoplasmic vesicle lumen | 0.319489 | 0.431495 | 0.369702 | S100A8 |
| CC | GO:0031983 | vesicle lumen | 0.321113 | 0.431495 | 0.369702 | S100A8 |
| CC | GO:1902495 | transmembrane transporter complex | 0.36052 | 0.440917 | 0.377774 | HSPA2 |
| CC | GO:0005769 | early endosome | 0.369647 | 0.440917 | 0.377774 | SLC35D3 |
| CC | GO:1990351 | transporter complex | 0.377157 | 0.440917 | 0.377774 | HSPA2 |
| CC | GO:0005819 | spindle | 0.379393 | 0.440917 | 0.377774 | HSPA2 |
| CC | GO:0030133 | transport vesicle | 0.379393 | 0.440917 | 0.377774 | CPE |
| CC | GO:0030055 | cell-substrate junction | 0.398456 | 0.446368 | 0.382445 | HMCN1 |
| CC | GO:0005759 | mitochondrial matrix | 0.430133 | 0.446368 | 0.382445 | SOD2 |
| CC | GO:0005635 | nuclear envelope | 0.434234 | 0.446368 | 0.382445 | PLA2G4A |
| CC | GO:0005667 | transcription regulator complex | 0.436952 | 0.446368 | 0.382445 | DACH1 |
| CC | GO:0005743 | mitochondrial inner membrane | 0.442351 | 0.446368 | 0.382445 | PLA2G4A |
| CC | GO:0005911 | cell-cell junction | 0.446368 | 0.446368 | 0.382445 | HMCN1 |
| MF | GO:0045236 | CXCR chemokine receptor binding | 0.000226 | 0.021439 | 0.013303 | CXCL10/CXCL11 |
| MF | GO:0008009 | chemokine activity | 0.001694 | 0.053899 | 0.033444 | CXCL10/CXCL11 |
| MF | GO:0048018 | receptor ligand activity | 0.002915 | 0.053899 | 0.033444 | CXCL10/CXCL11/RETNLB/TNFSF9 |
| MF | GO:0005125 | cytokine activity | 0.003011 | 0.053899 | 0.033444 | CXCL10/CXCL11/TNFSF9 |
| MF | GO:0030546 | signaling receptor activator activity | 0.003068 | 0.053899 | 0.033444 | CXCL10/CXCL11/RETNLB/TNFSF9 |
| MF | GO:0042379 | chemokine receptor binding | 0.003521 | 0.053899 | 0.033444 | CXCL10/CXCL11 |
| MF | GO:0030246 | carbohydrate binding | 0.004446 | 0.053899 | 0.033444 | CD69/CLC/CLEC4A |
| MF | GO:0005126 | cytokine receptor binding | 0.004539 | 0.053899 | 0.033444 | CXCL10/CXCL11/TNFSF9 |
| MF | GO:0015165 | pyrimidine nucleotide-sugar transmembrane transporter activity | 0.012426 | 0.092919 | 0.057656 | SLC35D3 |
| MF | GO:0018455 | alcohol dehydrogenase [NAD(P)+] activity | 0.012426 | 0.092919 | 0.057656 | ADH1C |
| MF | GO:0050786 | RAGE receptor binding | 0.012426 | 0.092919 | 0.057656 | S100A8 |
| MF | GO:0008603 | cAMP-dependent protein kinase regulator activity | 0.013661 | 0.092919 | 0.057656 | CXCL10 |
| MF | GO:0102545 | phosphatidyl phospholipase B activity | 0.013661 | 0.092919 | 0.057656 | PLA2G4A |
| MF | GO:0005338 | nucleotide-sugar transmembrane transporter activity | 0.014894 | 0.092919 | 0.057656 | SLC35D3 |
| MF | GO:0035325 | Toll-like receptor binding | 0.014894 | 0.092919 | 0.057656 | S100A8 |
| MF | GO:0008201 | heparin binding | 0.01847 | 0.092919 | 0.057656 | CXCL10/CXCL11 |
| MF | GO:0036041 | long-chain fatty acid binding | 0.018584 | 0.092919 | 0.057656 | S100A8 |
| MF | GO:0042043 | neurexin family protein binding | 0.018584 | 0.092919 | 0.057656 | CPE |
| MF | GO:0097153 | cysteine-type endopeptidase activity involved in apoptotic process | 0.018584 | 0.092919 | 0.057656 | CLC |
| MF | GO:0047498 | calcium-dependent phospholipase A2 activity | 0.019811 | 0.094102 | 0.05839 | PLA2G4A |
| MF | GO:0008237 | metallopeptidase activity | 0.023025 | 0.096997 | 0.060186 | CPE/MMP1 |
| MF | GO:0004745 | NAD-retinol dehydrogenase activity | 0.023483 | 0.096997 | 0.060186 | ADH1C |
| MF | GO:0010314 | phosphatidylinositol-5-phosphate binding | 0.023483 | 0.096997 | 0.060186 | PLA2G4A |
| MF | GO:0097001 | ceramide binding | 0.024705 | 0.097789 | 0.060678 | PLA2G4A |
| MF | GO:0005537 | mannose binding | 0.027143 | 0.103143 | 0.064 | CLEC4A |
| MF | GO:0004622 | lysophospholipase activity | 0.029575 | 0.105778 | 0.065635 | PLA2G4A |
| MF | GO:0005539 | glycosaminoglycan binding | 0.034155 | 0.105778 | 0.065635 | CXCL10/CXCL11 |
| MF | GO:0004181 | metallocarboxypeptidase activity | 0.03563 | 0.105778 | 0.065635 | CPE |
| MF | GO:0046625 | sphingolipid binding | 0.03563 | 0.105778 | 0.065635 | PLA2G4A |
| MF | GO:0051787 | misfolded protein binding | 0.03563 | 0.105778 | 0.065635 | HSPA2 |
| MF | GO:0051861 | glycolipid binding | 0.03563 | 0.105778 | 0.065635 | HSPA2 |
| MF | GO:0070273 | phosphatidylinositol-4-phosphate binding | 0.03563 | 0.105778 | 0.065635 | PLA2G4A |
| MF | GO:0005164 | tumor necrosis factor receptor binding | 0.038042 | 0.109516 | 0.067954 | TNFSF9 |
| MF | GO:0004623 | phospholipase A2 activity | 0.041649 | 0.113094 | 0.070174 | PLA2G4A |
| MF | GO:0097718 | disordered domain specific binding | 0.042849 | 0.113094 | 0.070174 | HSPA2 |
| MF | GO:1901681 | sulfur compound binding | 0.043404 | 0.113094 | 0.070174 | CXCL10/CXCL11 |
| MF | GO:0042056 | chemoattractant activity | 0.044047 | 0.113094 | 0.070174 | CXCL10 |
| MF | GO:0019825 | oxygen binding | 0.047633 | 0.118374 | 0.073451 | SOD2 |
| MF | GO:0001664 | G protein-coupled receptor binding | 0.049726 | 0.118374 | 0.073451 | CXCL10/CXCL11 |
| MF | GO:0044183 | protein folding chaperone | 0.052393 | 0.118374 | 0.073451 | HSPA2 |
| MF | GO:0048156 | tau protein binding | 0.052393 | 0.118374 | 0.073451 | HSPA2 |
| MF | GO:0004180 | carboxypeptidase activity | 0.05358 | 0.118374 | 0.073451 | CPE |
| MF | GO:0032266 | phosphatidylinositol-3-phosphate binding | 0.05358 | 0.118374 | 0.073451 | PLA2G4A |
| MF | GO:0005504 | fatty acid binding | 0.059492 | 0.1242 | 0.077066 | S100A8 |
| MF | GO:0032813 | tumor necrosis factor receptor superfamily binding | 0.059492 | 0.1242 | 0.077066 | TNFSF9 |
| MF | GO:0015932 | nucleobase-containing compound transmembrane transporter activity | 0.06067 | 0.1242 | 0.077066 | SLC35D3 |
| MF | GO:0016887 | ATP hydrolysis activity | 0.061621 | 0.1242 | 0.077066 | HSPA2/HSPA4L |
| MF | GO:0008374 | O-acyltransferase activity | 0.064196 | 0.1242 | 0.077066 | PLA2G4A |
| MF | GO:1901505 | carbohydrate derivative transmembrane transporter activity | 0.064196 | 0.1242 | 0.077066 | SLC35D3 |
| MF | GO:0005544 | calcium-dependent phospholipid binding | 0.065368 | 0.1242 | 0.077066 | PLA2G4A |
| MF | GO:0030145 | manganese ion binding | 0.078174 | 0.145617 | 0.090355 | SOD2 |
| MF | GO:0048029 | monosaccharide binding | 0.085087 | 0.154572 | 0.095911 | CLEC4A |
| MF | GO:0008235 | metalloexopeptidase activity | 0.086235 | 0.154572 | 0.095911 | CPE |
| MF | GO:0033293 | monocarboxylic acid binding | 0.0965 | 0.169768 | 0.105341 | S100A8 |
| MF | GO:0004175 | endopeptidase activity | 0.100625 | 0.171385 | 0.106344 | CLC/MMP1 |
| MF | GO:0016209 | antioxidant activity | 0.101027 | 0.171385 | 0.106344 | SOD2 |
| MF | GO:0048306 | calcium-dependent protein binding | 0.103282 | 0.172137 | 0.106811 | S100A8 |
| MF | GO:0015297 | antiporter activity | 0.105532 | 0.172854 | 0.107256 | SLC35D3 |
| MF | GO:0008238 | exopeptidase activity | 0.120025 | 0.190673 | 0.118312 | CPE |
| MF | GO:0031490 | chromatin DNA binding | 0.123337 | 0.190673 | 0.118312 | ZIC2 |
| MF | GO:0004620 | phospholipase activity | 0.124439 | 0.190673 | 0.118312 | PLA2G4A |
| MF | GO:0051087 | chaperone binding | 0.124439 | 0.190673 | 0.118312 | HSPA2 |
| MF | GO:0004222 | metalloendopeptidase activity | 0.129927 | 0.195921 | 0.121569 | MMP1 |
| MF | GO:0004197 | cysteine-type endopeptidase activity | 0.139722 | 0.202709 | 0.125781 | CLC |
| MF | GO:0051082 | unfolded protein binding | 0.140804 | 0.202709 | 0.125781 | HSPA2 |
| MF | GO:0005179 | hormone activity | 0.141884 | 0.202709 | 0.125781 | RETNLB |
| MF | GO:0031072 | heat shock protein binding | 0.142963 | 0.202709 | 0.125781 | HSPA2 |
| MF | GO:0016616 | oxidoreductase activity, acting on the CH-OH group of donors, NAD or NADP as acceptor | 0.14834 | 0.207185 | 0.128558 | ADH1C |
| MF | GO:0016298 | lipase activity | 0.150482 | 0.207185 | 0.128558 | PLA2G4A |
| MF | GO:0016614 | oxidoreductase activity, acting on CH-OH group of donors | 0.161113 | 0.216986 | 0.13464 | ADH1C |
| MF | GO:0043177 | organic acid binding | 0.162169 | 0.216986 | 0.13464 | S100A8 |
| MF | GO:0052689 | carboxylic ester hydrolase activity | 0.170572 | 0.22506 | 0.139649 | PLA2G4A |
| MF | GO:0005201 | extracellular matrix structural constituent | 0.194283 | 0.24666 | 0.153052 | HMCN1 |
| MF | GO:0031406 | carboxylic acid binding | 0.195299 | 0.24666 | 0.153052 | S100A8 |
| MF | GO:0004252 | serine-type endopeptidase activity | 0.196314 | 0.24666 | 0.153052 | MMP1 |
| MF | GO:1901981 | phosphatidylinositol phosphate binding | 0.197328 | 0.24666 | 0.153052 | PLA2G4A |
| MF | GO:0008234 | cysteine-type peptidase activity | 0.200361 | 0.247199 | 0.153387 | CLC |
| MF | GO:0008514 | organic anion transmembrane transporter activity | 0.208398 | 0.253817 | 0.157493 | SLC35D3 |
| MF | GO:0008236 | serine-type peptidase activity | 0.213381 | 0.256597 | 0.159218 | MMP1 |
| MF | GO:0017171 | serine hydrolase activity | 0.217346 | 0.258098 | 0.160149 | MMP1 |
| MF | GO:0019887 | protein kinase regulator activity | 0.229126 | 0.268728 | 0.166746 | CXCL10 |
| MF | GO:0016747 | acyltransferase activity, transferring groups other than amino-acyl groups | 0.239776 | 0.277789 | 0.172368 | PLA2G4A |
| MF | GO:0019207 | kinase regulator activity | 0.255958 | 0.292964 | 0.181784 | CXCL10 |
| MF | GO:0015291 | secondary active transmembrane transporter activity | 0.260655 | 0.294788 | 0.182916 | SLC35D3 |
| MF | GO:0016746 | acyltransferase activity | 0.264391 | 0.295496 | 0.183355 | PLA2G4A |
| MF | GO:0008017 | microtubule binding | 0.290047 | 0.317701 | 0.197133 | S100A8 |
| MF | GO:0035091 | phosphatidylinositol binding | 0.290948 | 0.317701 | 0.197133 | PLA2G4A |
| MF | GO:0008509 | anion transmembrane transporter activity | 0.327788 | 0.353862 | 0.219571 | SLC35D3 |
| MF | GO:0001227 | DNA-binding transcription repressor activity, RNA polymerase II-specific | 0.332899 | 0.354968 | 0.220257 | DACH1 |
| MF | GO:0001217 | DNA-binding transcription repressor activity | 0.336285 | 0.354968 | 0.220257 | DACH1 |
| MF | GO:0046982 | protein heterodimerization activity | 0.342173 | 0.357213 | 0.22165 | BCL2A1 |
| MF | GO:0015631 | tubulin binding | 0.378049 | 0.390377 | 0.242229 | S100A8 |
| MF | GO:0033218 | amide binding | 0.398362 | 0.404152 | 0.250776 | PLA2G4A |
| MF | GO:0022804 | active transmembrane transporter activity | 0.399897 | 0.404152 | 0.250776 | SLC35D3 |
| MF | GO:0005543 | phospholipid binding | 0.446403 | 0.446403 | 0.276992 | PLA2G4A |
