# Supplementary material for: Prognostic Assessment of Oxidative Stress-Related Genes in Colorectal Cancer and New Insights into Tumor Immunity
Source: Oxid Med Cell Longev. 2022 Oct 15;2022:2518340. doi: 10.1155/2022/2518340 (PMC9590115; doi:10.1155/2022/2518340)
Supplement: Supplementary 5 — Supplement 5: Table 4: KEGG enrichment analysis. [file 2518340.f5.docx]

KEGG enrichment analysis

| ID | Description | pvalue | p.adjust | qvalue |
| --- | --- | --- | --- | --- |
| hsa04657 | IL-17 signaling pathway | 0.000389 | 0.026482 | 0.023776 |
| hsa04213 | Longevity regulating pathway - multiple species | 0.004198 | 0.096176 | 0.08635 |
| hsa05417 | Lipid and atherosclerosis | 0.004243 | 0.096176 | 0.08635 |
| hsa04060 | Cytokine-cytokine receptor interaction | 0.010215 | 0.129778 | 0.116519 |
| hsa04061 | Viral protein interaction with cytokine and cytokine receptor | 0.010621 | 0.129778 | 0.116519 |
| hsa04620 | Toll-like receptor signaling pathway | 0.011451 | 0.129778 | 0.116519 |
| hsa04141 | Protein processing in endoplasmic reticulum | 0.029264 | 0.241246 | 0.216599 |
| hsa04062 | Chemokine signaling pathway | 0.036229 | 0.241246 | 0.216599 |
| hsa00592 | alpha-Linolenic acid metabolism | 0.039133 | 0.241246 | 0.216599 |
| hsa00591 | Linoleic acid metabolism | 0.045261 | 0.241246 | 0.216599 |
| hsa05171 | Coronavirus disease - COVID-19 | 0.051085 | 0.241246 | 0.216599 |
| hsa00350 | Tyrosine metabolism | 0.055899 | 0.241246 | 0.216599 |
| hsa05219 | Bladder cancer | 0.063431 | 0.241246 | 0.216599 |
| hsa00071 | Fatty acid degradation | 0.066428 | 0.241246 | 0.216599 |
| hsa04940 | Type I diabetes mellitus | 0.066428 | 0.241246 | 0.216599 |
| hsa00620 | Pyruvate metabolism | 0.072395 | 0.241246 | 0.216599 |
| hsa00565 | Ether lipid metabolism | 0.075366 | 0.241246 | 0.216599 |
| hsa04010 | MAPK signaling pathway | 0.07769 | 0.241246 | 0.216599 |
| hsa04913 | Ovarian steroidogenesis | 0.078327 | 0.241246 | 0.216599 |
| hsa05134 | Legionellosis | 0.08716 | 0.241246 | 0.216599 |
| hsa04370 | VEGF signaling pathway | 0.090087 | 0.241246 | 0.216599 |
| hsa04730 | Long-term depression | 0.091547 | 0.241246 | 0.216599 |
| hsa00590 | Arachidonic acid metabolism | 0.093005 | 0.241246 | 0.216599 |
| hsa04623 | Cytosolic DNA-sensing pathway | 0.095915 | 0.241246 | 0.216599 |
| hsa00010 | Glycolysis / Gluconeogenesis | 0.101708 | 0.241246 | 0.216599 |
| hsa05221 | Acute myeloid leukemia | 0.101708 | 0.241246 | 0.216599 |
| hsa00830 | Retinol metabolism | 0.103151 | 0.241246 | 0.216599 |
| hsa04664 | Fc epsilon RI signaling pathway | 0.103151 | 0.241246 | 0.216599 |
| hsa04622 | RIG-I-like receptor signaling pathway | 0.10603 | 0.241246 | 0.216599 |
| hsa00982 | Drug metabolism - cytochrome P450 | 0.108901 | 0.241246 | 0.216599 |
| hsa03320 | PPAR signaling pathway | 0.113192 | 0.241246 | 0.216599 |
| hsa00980 | Metabolism of xenobiotics by cytochrome P450 | 0.117463 | 0.241246 | 0.216599 |
| hsa04612 | Antigen processing and presentation | 0.117463 | 0.241246 | 0.216599 |
| hsa04146 | Peroxisome | 0.123129 | 0.241246 | 0.216599 |
| hsa04211 | Longevity regulating pathway | 0.132963 | 0.241246 | 0.216599 |
| hsa04912 | GnRH signaling pathway | 0.138537 | 0.241246 | 0.216599 |
| hsa05323 | Rheumatoid arthritis | 0.138537 | 0.241246 | 0.216599 |
| hsa04666 | Fc gamma R-mediated phagocytosis | 0.144077 | 0.241246 | 0.216599 |
| hsa00564 | Glycerophospholipid metabolism | 0.145457 | 0.241246 | 0.216599 |
| hsa04750 | Inflammatory mediator regulation of TRP channels | 0.145457 | 0.241246 | 0.216599 |
| hsa05231 | Choline metabolism in cancer | 0.145457 | 0.241246 | 0.216599 |
| hsa04064 | NF-kappa B signaling pathway | 0.153694 | 0.248838 | 0.223415 |
| hsa04668 | TNF signaling pathway | 0.164563 | 0.249243 | 0.223778 |
| hsa05145 | Toxoplasmosis | 0.164563 | 0.249243 | 0.223778 |
| hsa04724 | Glutamatergic synapse | 0.16726 | 0.249243 | 0.223778 |
| hsa04726 | Serotonergic synapse | 0.168605 | 0.249243 | 0.223778 |
| hsa04611 | Platelet activation | 0.180625 | 0.25699 | 0.230734 |
| hsa04926 | Relaxin signaling pathway | 0.187233 | 0.25699 | 0.230734 |
| hsa04068 | FoxO signaling pathway | 0.189862 | 0.25699 | 0.230734 |
| hsa04270 | Vascular smooth muscle contraction | 0.193791 | 0.25699 | 0.230734 |
| hsa04210 | Apoptosis | 0.196401 | 0.25699 | 0.230734 |
| hsa04915 | Estrogen signaling pathway | 0.199003 | 0.25699 | 0.230734 |
| hsa05162 | Measles | 0.200301 | 0.25699 | 0.230734 |
| hsa04936 | Alcoholic liver disease | 0.204183 | 0.25712 | 0.230851 |
| hsa03040 | Spliceosome | 0.210616 | 0.257303 | 0.231015 |
| hsa04072 | Phospholipase D signaling pathway | 0.211896 | 0.257303 | 0.231015 |
| hsa04921 | Oxytocin signaling pathway | 0.21954 | 0.26031 | 0.233715 |
| hsa05160 | Hepatitis C | 0.223336 | 0.26031 | 0.233715 |
| hsa04217 | Necroptosis | 0.225858 | 0.26031 | 0.233715 |
| hsa05164 | Influenza A | 0.240828 | 0.272938 | 0.245053 |
| hsa05202 | Transcriptional misregulation in cancer | 0.267581 | 0.298286 | 0.267811 |
| hsa05169 | Epstein-Barr virus infection | 0.278272 | 0.305201 | 0.27402 |
| hsa04024 | cAMP signaling pathway | 0.300371 | 0.321577 | 0.288722 |
| hsa05208 | Chemical carcinogenesis - reactive oxygen species | 0.302661 | 0.321577 | 0.288722 |
| hsa04014 | Ras signaling pathway | 0.316253 | 0.33085 | 0.297048 |
| hsa04144 | Endocytosis | 0.333996 | 0.344118 | 0.30896 |
| hsa05020 | Prion disease | 0.3577 | 0.363039 | 0.325948 |
| hsa05016 | Huntington disease | 0.391798 | 0.391798 | 0.351769 |
